# Exposure to diesel exhaust particles results in altered lung microbial profiles, associated with increased reactive oxygen species/reactive nitrogen species and inflammation, in C57Bl/6 wildtype mice on a high-fat diet

**DOI:** 10.1186/s12989-020-00393-9

**Published:** 2021-01-08

**Authors:** Sarah Daniel, Danielle Phillippi, Leah J. Schneider, Kayla N. Nguyen, Julie Mirpuri, Amie K. Lund

**Affiliations:** 1 Advanced Environmental Research Institute, Department of Biological Sciences, University of North Texas, EESAT – 215, 1704 W. Mulberry, Denton, TX 76201 USA; 2 Division of Neonatal-Perinatal Medicine, Department of Pediatrics, UT Southwestern Medical Center, Dallas, TX 75390 USA

**Keywords:** Diesel particulate matter, Lung microbiome, Inflammation, Probiotics, Reactive oxygen species, Reactive nitrogen species

## Abstract

**Background:**

Exposure to traffic-generated emissions is associated with the development and exacerbation of inflammatory lung disorders such as chronic obstructive pulmonary disorder (COPD) and idiopathic pulmonary fibrosis (IPF). Although many lung diseases show an expansion of Proteobacteria, the role of traffic-generated particulate matter pollutants on the lung microbiota has not been well-characterized. Thus, we investigated the hypothesis that exposure to diesel exhaust particles (DEP) can alter commensal lung microbiota, thereby promoting alterations in the lung’s immune and inflammatory responses. We aimed to understand whether diet might also contribute to the alteration of the commensal lung microbiome, either alone or related to exposure. To do this, we used male C57Bl/6 mice (4–6-week-old) on either regular chow (LF) or high-fat (HF) diet (45% kcal fat), randomly assigned to be exposed via oropharyngeal aspiration to 35 μg DEP, suspended in 35 μl 0.9% sterile saline or sterile saline only (control) twice a week for 30 days. A separate group of study animals on the HF diet was concurrently treated with 0.3 g/day of Winclove Ecologic® Barrier probiotics in their drinking water throughout the study.

**Results:**

Our results show that DEP-exposure increases lung tumor necrosis factor (TNF)-α, interleukin (IL)-10, Toll-like receptor (TLR)-2, TLR-4, and the nuclear factor kappa B (NF-κB) histologically and by RT-qPCR, as well as Immunoglobulin A (IgA) and Immunoglobulin G (IgG) in the bronchoalveolar lavage fluid (BALF), as quantified by ELISA. We also observed an increase in macrophage infiltration and peroxynitrite, a marker of reactive oxygen species (ROS) + reactive nitrogen species (RNS), immunofluorescence staining in the lungs of DEP-exposed and HF-diet animals, which was further exacerbated by concurrent DEP-exposure and HF-diet consumption. Histological examinations revealed enhanced inflammation and collagen deposition in the lungs DEP-exposed mice, regardless of diet. We observed an expansion of Proteobacteria, by qPCR of bacterial 16S rRNA, in the BALF of DEP-exposed mice on the HF diet, which was diminished with probiotic-treatment.

**Conclusions:**

Our findings suggest that exposure to DEP causes persistent and sustained inflammation and bacterial alterations in a ROS-RNS mediated fashion, which is exacerbated by concurrent consumption of an HF diet.

**Supplementary Information:**

The online version contains supplementary material available at 10.1186/s12989-020-00393-9.

## Background

The lungs are the first organs within the human body to be continuously exposed to particulates within inhaled air. Several studies have described the toxicological implications of particulates within the lungs leading to pronounced inflammation and reduced lung function [1, 2]. There is increasing evidence suggesting that exposure to the particulate matter (PM) component of ambient air pollution, including that derived from traffic-generated sources, can contribute to or exacerbate lung disorders such as asthma, chronic obstructive pulmonary disorder (COPD), bronchitis, and idiopathic pulmonary fibrosis (IPF) [3, 4]. The incidence of these diseases is also higher in polluted regions, suggesting that PM air pollutants play a crucial role in either development or exacerbation of underlying lung conditions. Reactive oxygen species (ROS) have been reported to be an essential driving factor in the PM-mediated inflammatory response observed within the lungs [5]. PM_2.5_ exposure is also well documented to induce pulmonary inflammation, often characterized by the expression of several inflammatory markers, including tumor necrosis factor (TNF)-α, interleukin (IL)-1β, IL-6, and IL-10 [6, 7]. Ambient air pollution PM has also been shown to induce Toll-like receptor 2 (TLR2) expression and subsequent activation of the nuclear factor kappa B (NF-κB) transcription factor that accelerates the inflammatory response in both in vitro studies and in vivo exposures [8].

Although there has been extensive research in the observed inflammatory response associated with PM pollutant exposures, the involvement of the lung microbial communities has not yet been well-characterized. With emerging evidence on the presence of a commensal lung microbiome, it is becoming evident that these microbes are not mere bystanders, but they play a role in maintaining homeostasis, and their alterations may have detrimental outcomes in lung health.

The gut microbiome is associated with a wide variety of homeostatic functions, including nutrient absorption and immune system development [9, 10]. Alterations in the human gut microbiota have been associated with many gastrointestinal diseases, including inflammatory bowel disease [11]. On the other hand, the lung microbiome has not been explored extensively, nor are their functions in health understood. The lungs were always known to have bacterial colonization in diseases, but the discovery of the lung microbiome was delayed due to the long-held view that the lungs of healthy individuals were sterile [12]. This misconception was mainly due to sampling difficulties of the less abundant lung microbiome, but advances in sequencing techniques have revealed that the lower respiratory tract is replete with a wide variety of microorganisms in healthy individuals as well, and not only in individuals with lung diseases [13]. Murine lungs from different environmental conditions have been shown to host common microbial profiles [14]. However, the airway microbial composition varies considerably when compared to the GI tract [15]. The study by Ruane et al. shows that the airway microbes help prime lung dendritic cells induce the production of Immunoglobulin A (IgA), the predominant antibody at mucosal surfaces [16]. Germ-free mice were found to have a reduced ability for IgA production, predisposing them to irritants and microbial challenges. These mice were also found to have reduced mucus production, limiting their mucociliary defense functions [17].

The commensal flora arrives within the lungs from the oral cavity via microaspiration, [18]. However, studies suggest that the lungs may not host a constant microbial community due to continuous immigration and elimination by host immune defenses such as cough and mucociliary clearance [12, 18]. Despite these fluxes observed, most bacteria within the lungs belong to four major phyla – Firmicutes, Bacteroidetes, Proteobacteria, and Actinobacteria [19]. Unlike the gastrointestinal tract, nutrient availability within the lungs is sparse — mostly obtained from immunoglobulins, cytokines, and mucins. This decreased nutrient availability in the lung likely accounts for the low bacterial abundance ranging from 10^4^ to 10^8^ compared to the GI tract that harbors close to 10^12^ microbes [20]. Changes in the availability of these nutrients have a crucial impact on lung microbes. In many pulmonary disease states, there is an increase in mucus production, which favors the growth of certain opportunistic bacteria that can metabolize mucins and proliferate, especially bacteria belonging to the Proteobacteria phylum [21]. Proteobacteria are found to be present in higher amounts in many inflammatory diseases, mainly due to their unique abilities to utilize the byproducts of inflammation to proliferate [22].

The microbiome is influenced by several factors, including diet and environmental exposures [23, 24]. A large percentage of the Western world population consumes a diet rich in fats (typically> 30% fat), which has resulted in the epidemic of obesity [25]. Consumption of a high-fat (HF) diet has been shown to alter the intestinal microflora and increase baseline inflammation [26–28]. Although the gut and the lungs are distinct anatomical sites, recent studies show evidence of complex gut-lung cross-talk involving effector molecules that protect from environmental stimuli and infections at mucosal surfaces [29]. Dietary supplementation with probiotics has been shown to exert a wide range of beneficial effects with respect to reduced infections and attenuation of disease duration [30, 31]. Emerging studies show that probiotics exert pleiotropic effects – protective roles in the GI tract by competing with local pathogens, and indirect effects by changing the host immune system to noninflammatory tolerogenic patterns by inducing IL-10 producing T regulatory (T _reg_) cells [32]. The immunomodulatory effects of probiotics extend systemically and at distant mucosal sites, including the lungs, as observed by improvements in allergic airway diseases [33].

To date, the synergistic effects of both PM pollutants and HF diet consumption on the lung microbiota profiles and inflammation have not been explored. Understanding the interactions between environmental PM exposures, diet, microbiome, and the immune system is significant in the development of therapeutic alternatives in diseases. Although there has been significant interest in probiotic influences in disease states, fewer studies have investigated the effects of probiotics on air-pollution PM induced inflammation [34, 35]. Considering this gap in knowledge, we investigated whether exposure to diesel exhaust particles (DEP) results in inflammation and alterations in the lung microbiota in wild-type C57Bl/6 mice on either a standard mouse chow vs. HF diet.

## Results

### Exposure to DEP results in systemic and peri-bronchial inflammation, which is more pronounced with the consumption of the HF diet in C57Bl/6 wildtype mice

**Fig. 1:**
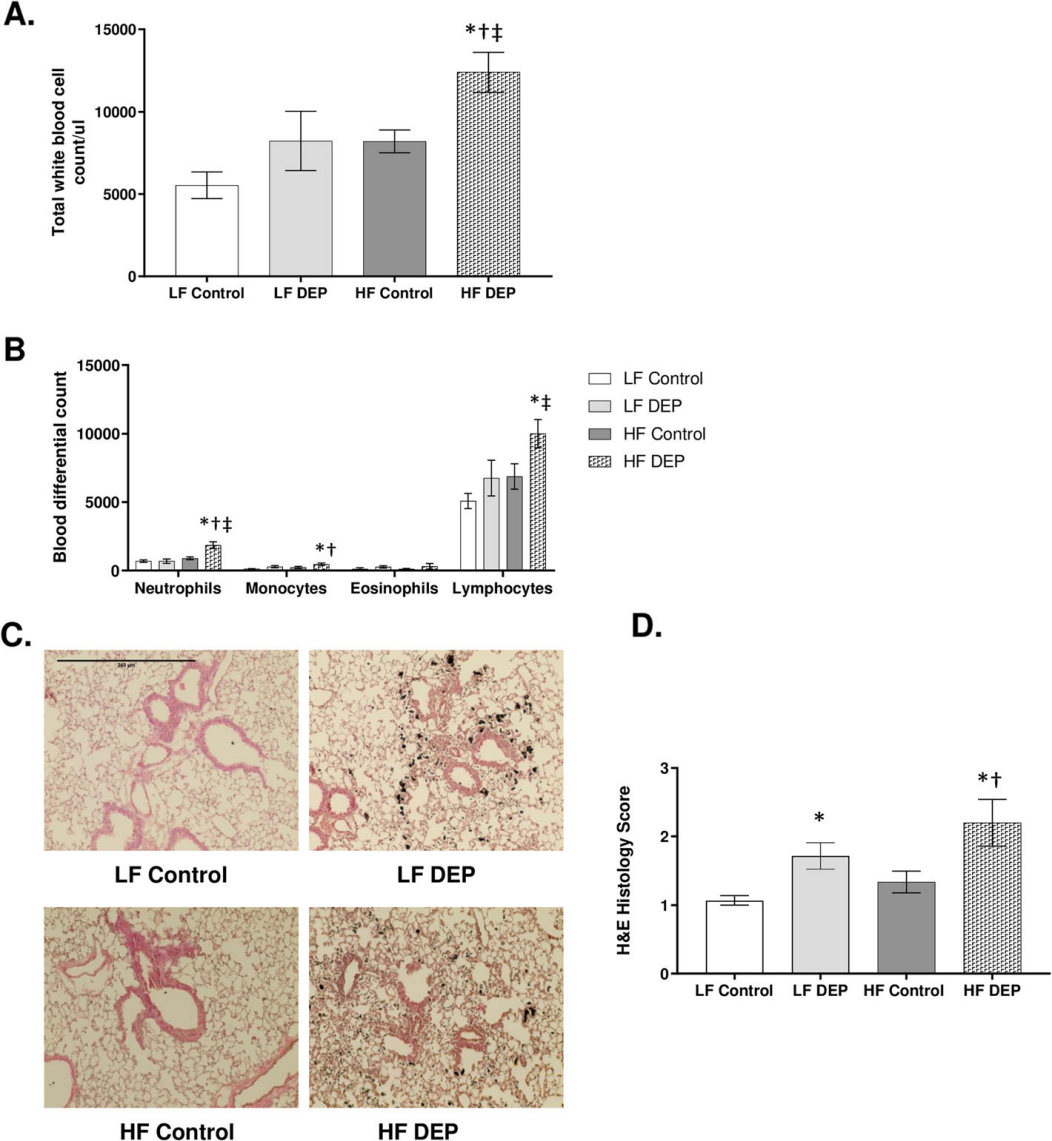
Exposure to diesel exhaust particles results in systemic and peri-bronchial inflammation. **a** Graphs representing total white blood cell count and **b** blood differential counts in 4–6 week-old male C57Bl/6 wildtype mice, on either control (LF) or high-fat (HF) diet exposed to either saline (control) or diesel exhaust particles (DEP – 35 μg PM) for 1 week. **c** Representative images of H&E staining of lung sections in control and exposed groups after 30 days of exposure. **d** Quantification of histological injury score in mice exposed to either DEP or saline. Images displayed are using 20X magnification. Scale bar = 240 μm. Data are depicted as mean ± SEM with *p < 0.05 compared to LF Control, †*p* < 0.05 compared to HF Control, ‡*p* < 0.05 compared to LF DEP by two way ANOVA

Consumption of an HF diet has been shown to not only increase susceptibility to metabolic diseases but also contribute to low-grade systemic inflammation [26]. To investigate whether subacute exposure to inhaled DEP and HF diet resulted in acute inflammation systemically, we quantified total white blood cells and performed differential blood counts after 5 days of DEP exposures. We observed that the total white blood cell counts were significantly higher in the HF DEP group when compared to the LF Control (*p* = 0.001), LF DEP (*p* = 0.030), and HF Control (*p* = 0.029) groups (Fig. 1a). The respective F values for the total white blood cell counts are: exposure = 7.539, diet = 7.402, exposure x diet interaction = 0.351. The differential counts for neutrophils were significantly higher in the HF DEP group when compared to LF Control, LF DEP, and HF Control (*p* < 0.001) with an F value of exposure = 17.490, diet = 8.503, exposure x diet interaction = 8.745 (Fig. 1b). Monocyte and lymphocyte counts were also higher in the HF DEP group compared to LF Control (*p* = 0.004), LF DEP (*p* = 0.033), and HF Control (*p* = 0.033), F = 7.097 for exposure (monocytes) and F = 4.965 for exposure (lymphocytes) (Fig. 1b). We did not detect any basophils in all the groups (data not shown) or any differences across the groups in eosinophils in the differential assessments. To assess whether inflammation persists within the lungs after 30 days of DEP exposure, we performed morphological examinations of the lung tissue sections. Compared to the controls, we observed significant peri-bronchial inflammation surrounding the bronchioles in mice exposed to DEP, regardless of the diet consumed (Fig. 1c). The histology scores for LF DEP were significantly higher than LF Control (*p* = 0.029). The inflammation was found to be more pronounced in the HF DEP group when compared to the LF Control (*p* < 0.001) and HF Control (*p* = 0.005), as indicated by the histology scores, F = 12.99 for exposure (Fig. 1d).

### Exposure to DEP results in increased levels of immunoglobulins in the BALF of C57Bl/6 mice

**Fig. 2:**
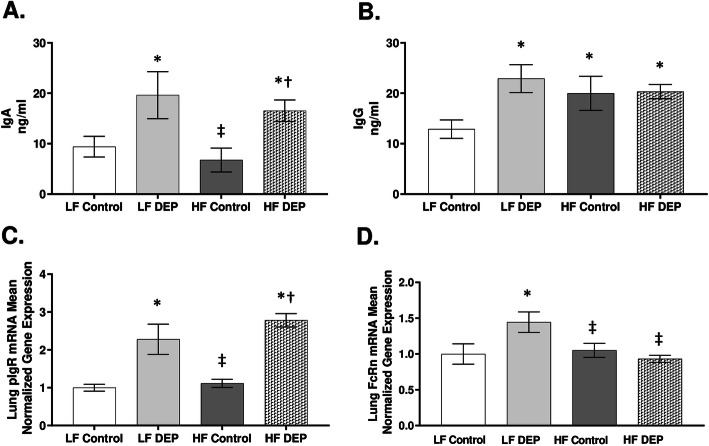
Exposure to DEP results in increased levels of immunoglobulins in the BALF. **a** Quantification of IgA (ng/ml) and **b** IgG (ng/ml), in the bronchoalveolar lavage fluid (BALF) of 4–6 week-old male C57Bl/6 wildtype mice, on either control (LF) or high-fat (HF) diet exposed to either saline (control) or diesel exhaust particles (DEP – 35 μg PM) twice a week for a total of 30 days, by ELISA. **c** Mean normalized gene expression of IgA receptor - pIgR and **d** IgG receptor – FcRn in lung tissues, as determined by RT-qPCR. Data are depicted as mean ± SEM with *p < 0.05 compared to LF Control, †*p* < 0.05 compared to HF Control, ‡*p* < 0.05 compared to LF DEP by two way ANOVA

IgA is the predominant immunoglobulin in mucosal secretions, and its main function is to provide the first line of defense by neutralizing toxins [36]. IgG, another important immune defense within airways, functions in close cooperation with IgA by fixing complement and opsonization [36]. To observe if DEP exposure alters the levels of IgA and IgG, we performed ELISA on the BALF obtained from these mice. We observed a significant increase in IgA regardless of the diet consumed. IgA was higher in LF DEP (*p* = 0.018) and HF DEP (*p* = 0.041) compared to LF Control and HF Control (*p* = 0.010, F = 13.670 for exposure (Fig. 2a). Compared to LF Control, IgG levels were also significantly elevated in the LF DEP (*p* = 0.004) and HF DEP (*p* = 0.027) (Fig. 2b). We also observed a significant increase in IgG in the HF Control group than LF Control (Fig. 2b, *p* = 0.045). The respective F values for BALF IgG levels are: exposure = 4.907, diet = 0.935, exposure x diet interaction = 4.286. To determine whether the upregulation of immunoglobulins in the lungs correlated to the expression of receptors responsible for transcytosis, we quantified the transcript expression of the polymeric IgA receptor (pIgR) responsible for the transcytosis of IgA as well as the neonatal Fc receptor (FcRn), the receptor for IgG transport into the airway lumen by RT-qPCR. We observed that the pIgR receptor expression was significantly elevated in the LF DEP and HF DEP groups compared to LF Control and HF Control (*p* < 0.001), F = 49.090 for exposure (Fig. 2c). FcRn expression was upregulated only in LF DEP compared with LF Control (*p* = 0.013) (Fig. 2d). We did not observe an increase in FcRn expression in HF Control and HF DEP groups. The F values for FcRn expression levels are: exposure = 2.090, diet = 4.199, exposure x diet interaction = 6.252.

### Exposure to DEP increases TNF-α and IL-10 expression in the lungs of C57Bl/6 mice

**Fig. 3:**
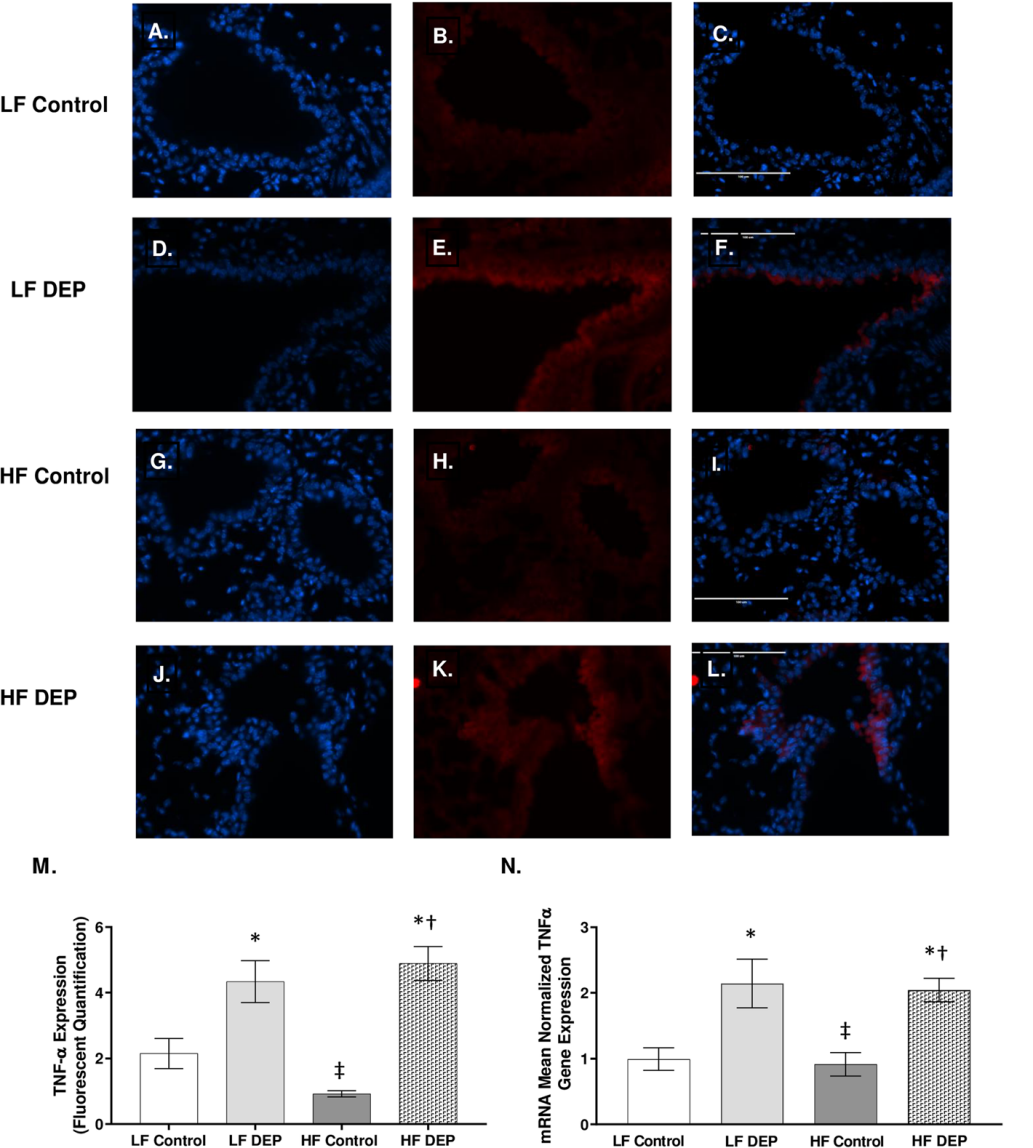
Exposure to diesel exhaust particles results in increased expression of TNF-α. Representative images of tumor necrosis factor (TNF)-α expression in the lungs of C57Bl/6 mice on a control (LF; **a-c**) or high-fat (HF; **g-i**) diet exposed to saline (control) or on a LF (**d-f**) or HF diet (**j-l**) exposed to diesel exhaust particles (DEP – 35 μg PM) twice a week for a total of 30 days. Red fluorescence indicates TNF-α expression, blue fluorescence is nuclear staining (Hoechst). Right panels (**c**, **f**, **i**, **l**) are merged figures of left (blue; **a**, **d**, **g**, **j**) and center (red; **b**, **e**, **h**, **k**) panels. **m** Graph of histology analysis of lung TNF-α fluorescence and **n** mean normalized gene expression of TNF-α mRNA transcript expression within the lungs, as determined by RT-qPCR. 40x magnification; scale bar = 100 μm. *p < 0.05 compared to LF Control, †*p* < 0.05 compared to HF Control, ‡*p* < 0.05 compared to LF DEP by two way ANOVA

**Fig. 4:**
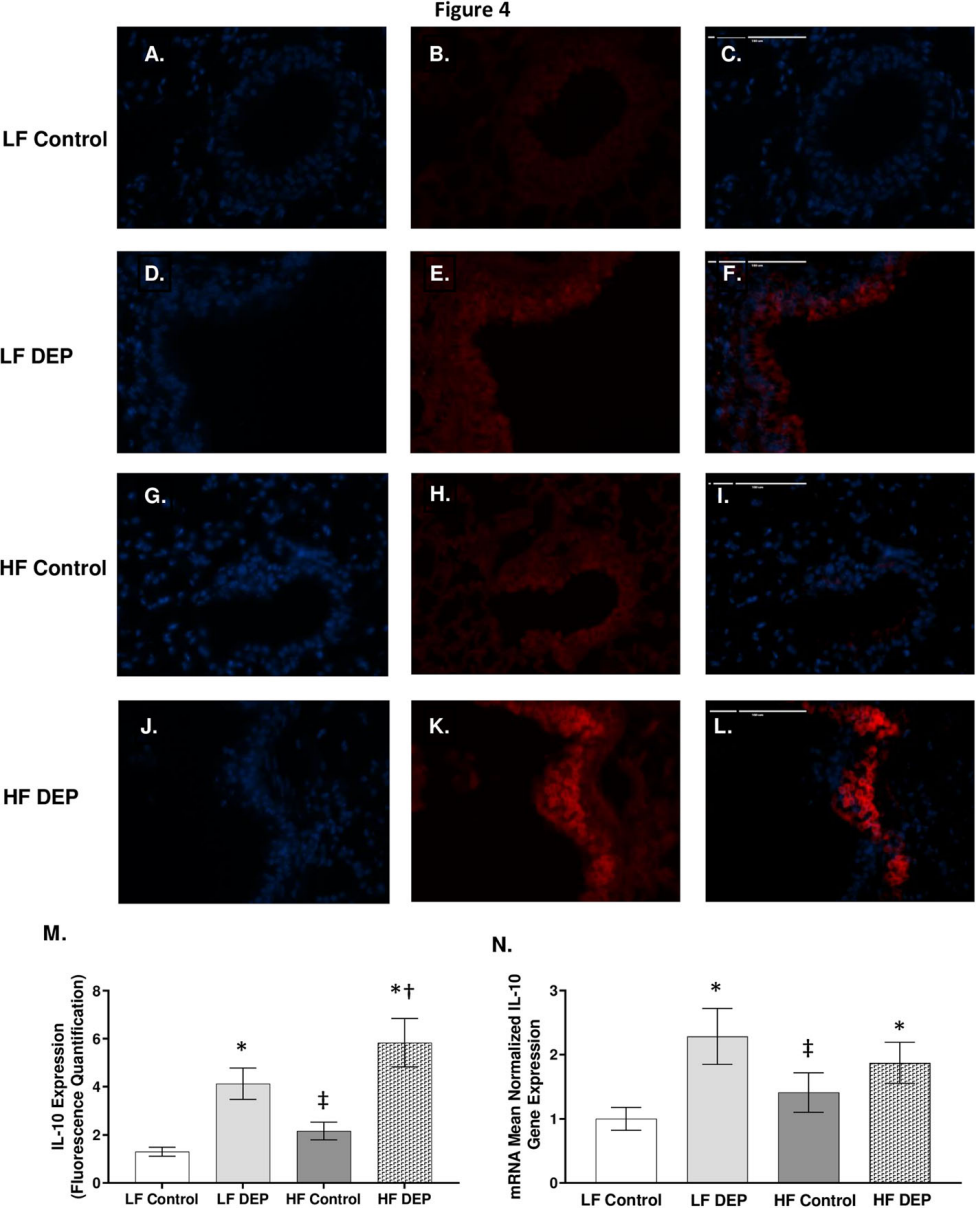
Exposure to diesel exhaust particles results in increased expression of IL-10. Representative images of Interleukin - 10 (IL-10) expression in the lungs of C57Bl/6 mice on a low-fat (LF; **a-c** or high-fat (HF; **g-i**) diet exposed to saline (control) or on a LF (**d-f**) or HF diet (**j-l**) exposed to diesel exhaust particles (DEP – 35 μg PM) twice a week for a total of 30 days. Red fluorescence indicates IL-10 expression, blue fluorescence is nuclear staining (Hoechst). Right panels (**c**, **f**, **i**, **l**) are merged figures of left (blue; **a**, **d**, **g**, **j**) and center (red; **b**, **e**, **h**, **k**) panels. **m** Graph of histology analysis of lung IL-10 fluorescence and **n** mean normalized gene expression of IL-10 mRNA transcript expression within the lung, as determined by RT-qPCR. 40x magnification, scale bar = 100 μm. *p < 0.05 compared to LF Control, †*p* < 0.05 compared to HF Control, ‡*p* < 0.05 compared to LF DEP by two way ANOVA

To investigate whether subchronic DEP-exposure results in persistent production of inflammatory cytokines, we analyzed the expression of pro-inflammatory cytokines - TNF-α, IL-1β, and IL-6. In comparison to the lungs from the LF (Fig. 3a-c) and HF (Fig. 3g-i) control animals, we observed a significant increase in TNF- α in the lungs from both the LF DEP (Fig. 3d-f, *p* = 0.001) and HF DEP (Fig. 3j-l, *p* < 0.001) groups, F = 42.48 for exposure, as quantified in Fig. 3m. Real-time RT-qPCR analysis showed similar trends in TNF-α mRNA transcript, with statistical increases being observed in the DEP-exposed lungs, independent of diet, compared to both the LF Control (Fig. 3n, *p* = 0.005) and HF control (Fig. 3n, *p* = 0.003; F = 22.590 for exposure). We did not detect any elevation in the expression of IL-1β and IL-6 (data not shown) across any of the groups. We suspected anti-inflammatory mediators to be present after a 30-day exposure. When we analyzed the expression of IL-10, an anti-inflammatory cytokine, we observed that in comparison to the lungs from the LF (Fig. 4a-c) and HF (Fig. 4g-i) control animals, there was a significant increase in IL-10 in the lungs from both the LF DEP (Fig. 4d-f, *p* = 0.002), and HF DEP (Fig. 4j-l, *p* < 0.001) groups, F = 25.960 for exposure, as quantified in Fig. 4m. When analyzed by real-time RT-qPCR, we observed a significant increase in the IL-10 mRNA transcript in the LF DEP (Fig. 4n, *p* = 0.006) and HF DEP (Fig. 4n, *p* = 0.039) exposed lungs, compared to the LF controls (Fig. 4n, F = 10.530 for exposure).

### Exposure to DEP results in elevated production of mucus in C57Bl/6 mice

**Fig. 5:**
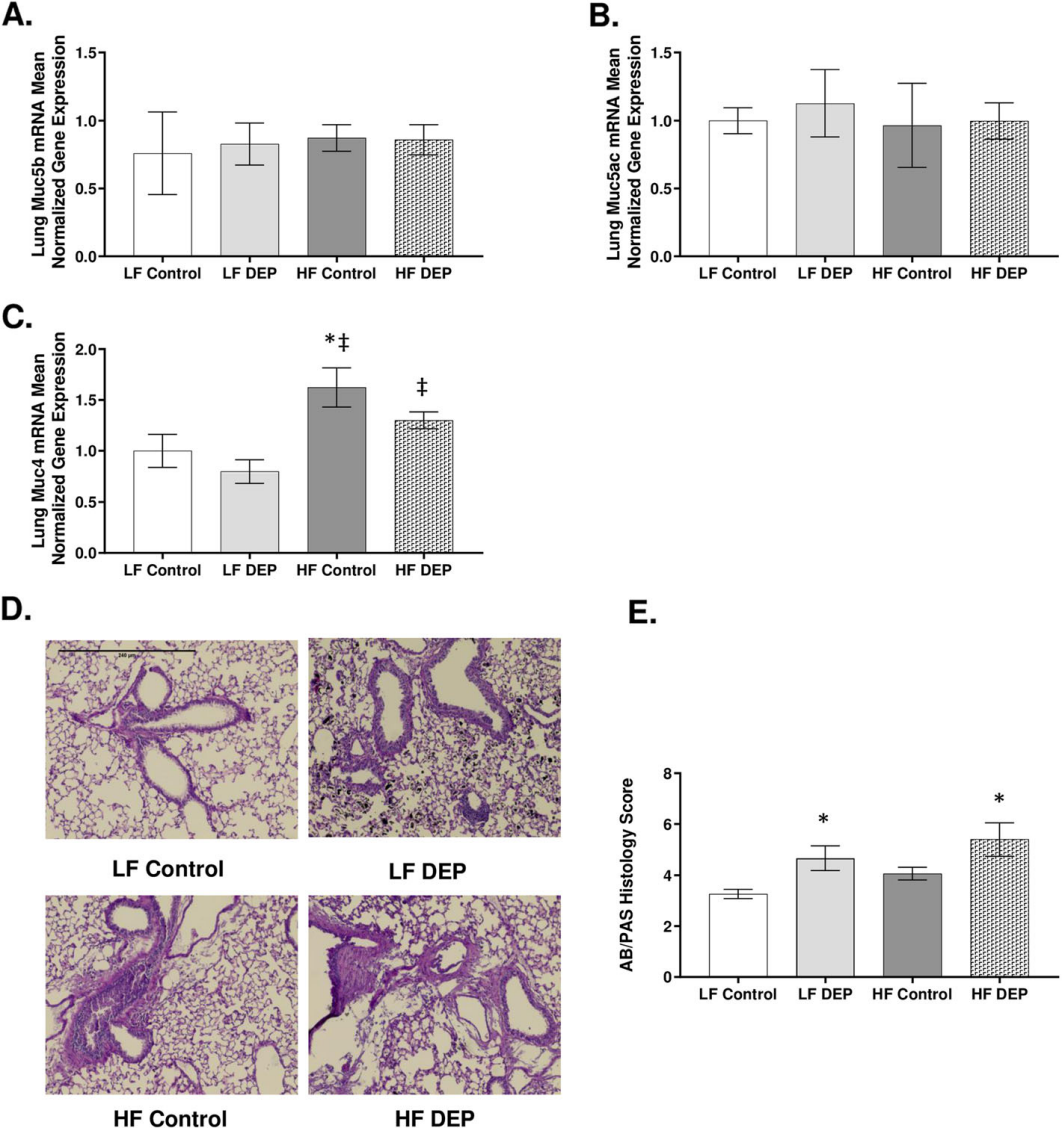
Exposure to diesel exhaust particles results in increased mucus production. **a** Mean normalized gene expression of Muc5b, **b** Muc5ac and **c** Muc4 mRNA transcript expression within the lungs of C57Bl/6 wildtype mice, as determined by RT-qPCR, on either control (LF) or high-fat (HF) diet, exposed to either saline (control) or diesel exhaust particles (DEP − 35 μg PM) twice a week for a total of 30 days. **d** Representative images of AB/PAS staining of lung sections and **e** quantification of histological mucus score. Images displayed are using 20X magnification, scale bar = 240 μm. Data are depicted as mean ± SEM with *p < 0.05 compared to LF Control, ‡*p* < 0.05 compared to LF DEP by two way ANOVA

A healthy mucus barrier maintained under physiological conditions is crucial in eliminating pathogens and irritants within the lungs [37]. When we investigated whether gel-bound mucins Muc5ac and Muc5b*,* which are considered integral components of the airway mucus, are elevated with DEP exposures, we observed no alteration at the transcript level after a 30-day exposure duration (Fig. 5a, b). Interestingly, we did observe a significant increase in the membrane-bound mucin Muc4 mRNA transcript in the lungs of the HF control compared to LF Control (*p* = 0.006) and LF DEP (*p* < 0.001). We also observed a significant increase in Muc4 in HF DEP compared to LF DEP (*p* = 0.02) (Fig. 5c). The F values for Muc4 levels are exposure = 3.316, diet = 15.200, exposure x diet interaction = 0.177; however, there was no alteration in expression of Muc4 mRNA transcript in the LF DEP group (Fig. 5c). When we stained for overall mucus produced within these lungs, compared to LF Control animals, we observed a significant increase in mucus production in the lungs of DEP exposed animals (Fig. 5d), as quantified in Fig. 5e (*p* = 0.011, F = 8.353 for exposure).

### Exposure to DEP results in an increased relative abundance of Proteobacteria in C57Bl/6 mice on the HF diet

**Fig. 6:**
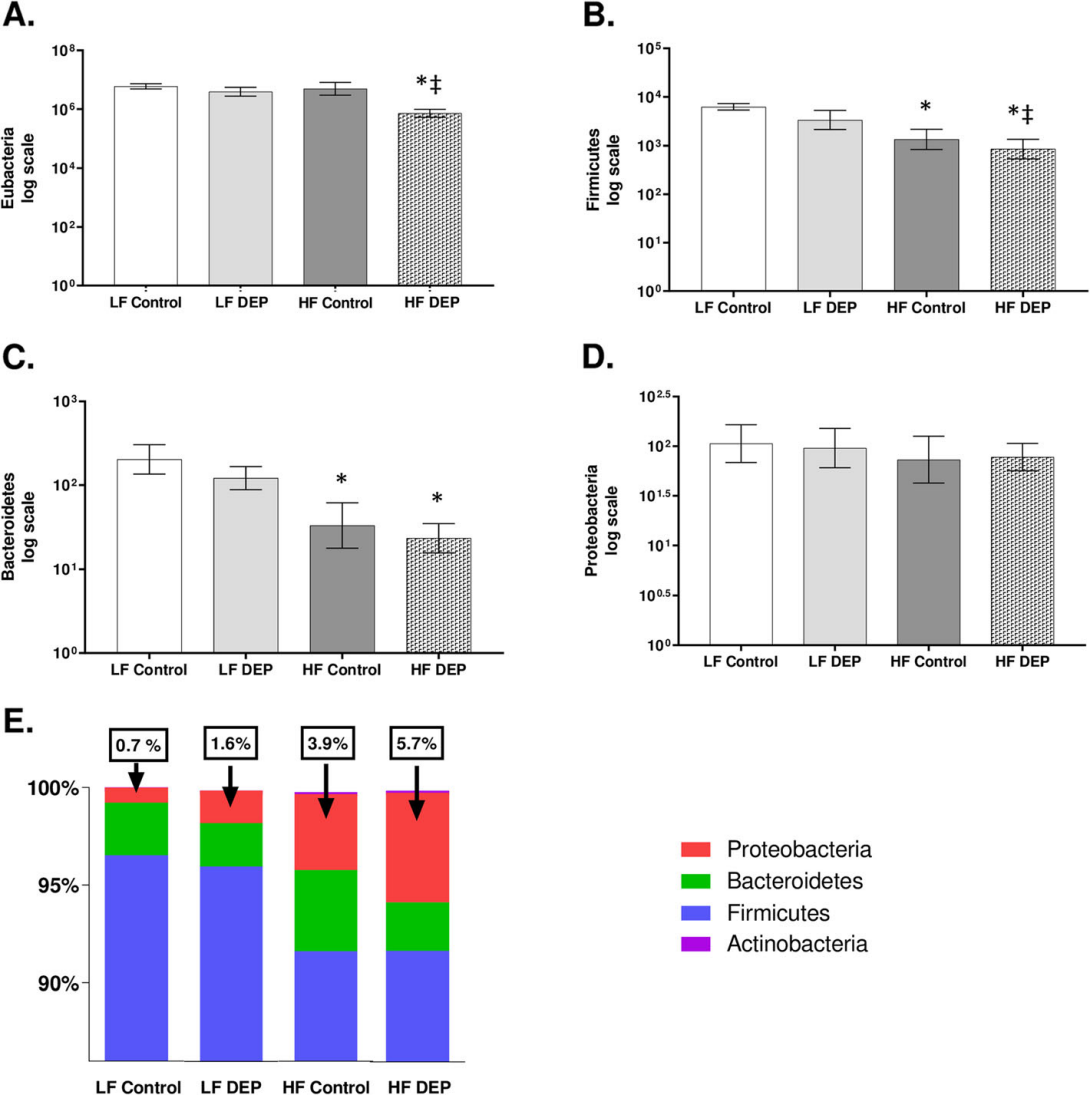
Exposure to diesel exhaust particles results in an increased relative abundance of Proteobacteria. **a** Quantification in log scale of (**a**) Eubacteria (total bacteria), **b** Firmicutes, **c** Bacteroidetes and **d** Proteobacteria within the lungs of C57Bl/6 wildtype mice on either control (LF) or high-fat (HF) diet exposed to either saline (control) or diesel exhaust particles (DEP – 35 μg PM) twice a week for a total of 30 days by qPCR. **e** 100% stacked columns representing the percentages of major lung phyla. Data are depicted as mean ± SEM with *p < 0.05 compared to LF Control, ‡*p* < 0.05 compared to LF DEP by two way ANOVA

Inflammation and mucus are now understood to increase the availability of nutrients that can be used by selective bacteria to proliferate and outcompete the others [38]. To investigate whether there are bacterial alterations within the lungs in response to DEP exposures and/or fat content in the diet, we performed qPCR of the 16srRNA region and quantified the total bacterial abundance. We quantified an average of 6.78 log copies of bacterial DNA in our LF Control groups (Fig. 6a). We observed that the total bacteria (Eubacteria) were decreasing in the HF DEP study groups compared to LF Control (*p* = 0.003) and compared to LF DEP (*p* = 0.017) (Fig. 6a) The respective F values for Eubacteria are: exposure = 2.490, diet = 9.228, exposure x diet interaction = 0.217. We also analyzed the quantitative abundance of individual phyla and observed that compared to LF Control, Firmicutes was significantly decreasing in the HF Control (*p* = 0.02) and HF DEP (*p* = 0.002). There was also a significant decrease in Firmicutes in the HF DEP group compared to the LF DEP (*p* = 0.017). (Fig. 6b). The F values for Firmicutes are: exposure = 1.645, diet = 12.040, exposure x diet interaction = 0.033. Compared to LF Control, there was a significant decrease in Bacteroidetes as well, in both the HF Control and the HF DEP group (*p* < 0.05) (Fig. 6c). The respective F values for Bacteroidetes are: exposure = 0.666, diet = 7.858, exposure x diet interaction = 0.356. There was no significant difference in Proteobacteria abundance noted in the lungs across any study groups (Fig. 6d, *p* = 0.070). However, when we measured the percentages of the individual phyla, contributing to the total bacterial abundance, for each of the study groups, we observed that the overall percentage of Proteobacteria was much higher in the lungs of the HF group, likely due to the significant decrease in both Firmicutes and Bacteroidetes (Fig. 6e).

### Exposure to DEP induces ROS-RNS production and increases macrophages within the lungs of C57Bl/6 mice

**Fig. 7:**
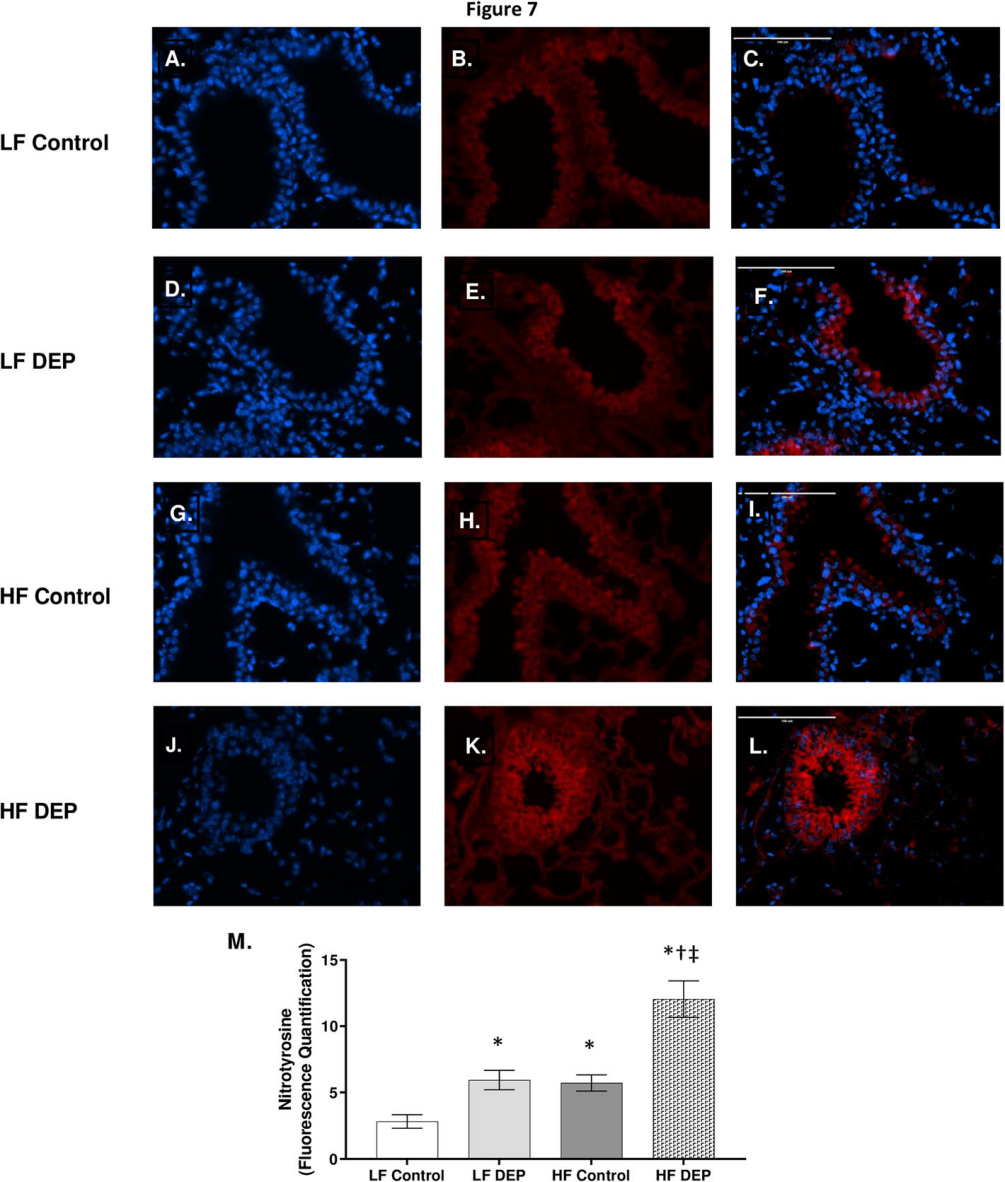
Exposure to diesel exhaust particles results in increased expression of nitrotyrosine. Representative images of nitrotyrosine expression in the lungs of C57Bl/6 mice on a control (LF; **a-c**) or high-fat (HF; **g-i**) diet exposed to saline (control) or on a LF (**d-f**) or HF diet (**j-l**) exposed to diesel exhaust particles (DEP – 35 μg PM) twice a week for a total of 30 days. Red fluorescence indicates nitrotyrosine expression, blue fluorescence is nuclear staining (Hoechst). Right panels (**c**, **f**, **i**, **l**) are merged figures of left (blue; **a**, **d**, **g**, **j**) and center (red; **b**, **e**, **h**, **k**) panels. **m** Graph of histology analysis of lung nitrotyrosine fluorescence. 40x magnification, scale bar = 100 μm. Data are depicted as mean ± SEM with *p < 0.05 compared to LF Control, †*p* < 0.05 compared to HF Control, ‡*p* < 0.05 compared to LF DEP by two way ANOVA

The generation of ROS and reactive nitrogen species (RNS) by macrophages is a well-understood mechanism of air-pollution mediated toxicity [5]. O_2_^−^ produced by ROS and NO_3_^−^ produced by RNS act as effectors against invading pathogens/pollutants and they are beneficial if these mechanisms are balanced by the physiological activity of antioxidant defenses. Oxidative stress occurs as a consequence of an increased generation of reactive species or reduced activity of antioxidant defenses [39]. To investigate the presence of these radicals we analyzed the combined effects of DEP and HF diet to induce extracellular ROS and RNS. We stained lung sections with nitrotyrosine that stains for peroxynitrite, a stable ROS + RNS product. When compared to LF (Fig. 7a-c) and HF (Fig. 7g-i) controls, we observed that nitrotyrosine was significantly expressed in the LF DEP (Fig. 7d-f, *p* =0.016) and HF DEP (Fig. 7j-l, *p* < 0.001) exposed groups, as quantified in Fig. 7m. We also observed a significant increase in nitrotyrosine in the HF Control groups compared to LF Controls (*p* = 0.013). The F values for nitrotyrosine are: exposure = 30.76, diet = 22.00, exposure x diet interaction = 4.412.

**Fig. 8:**
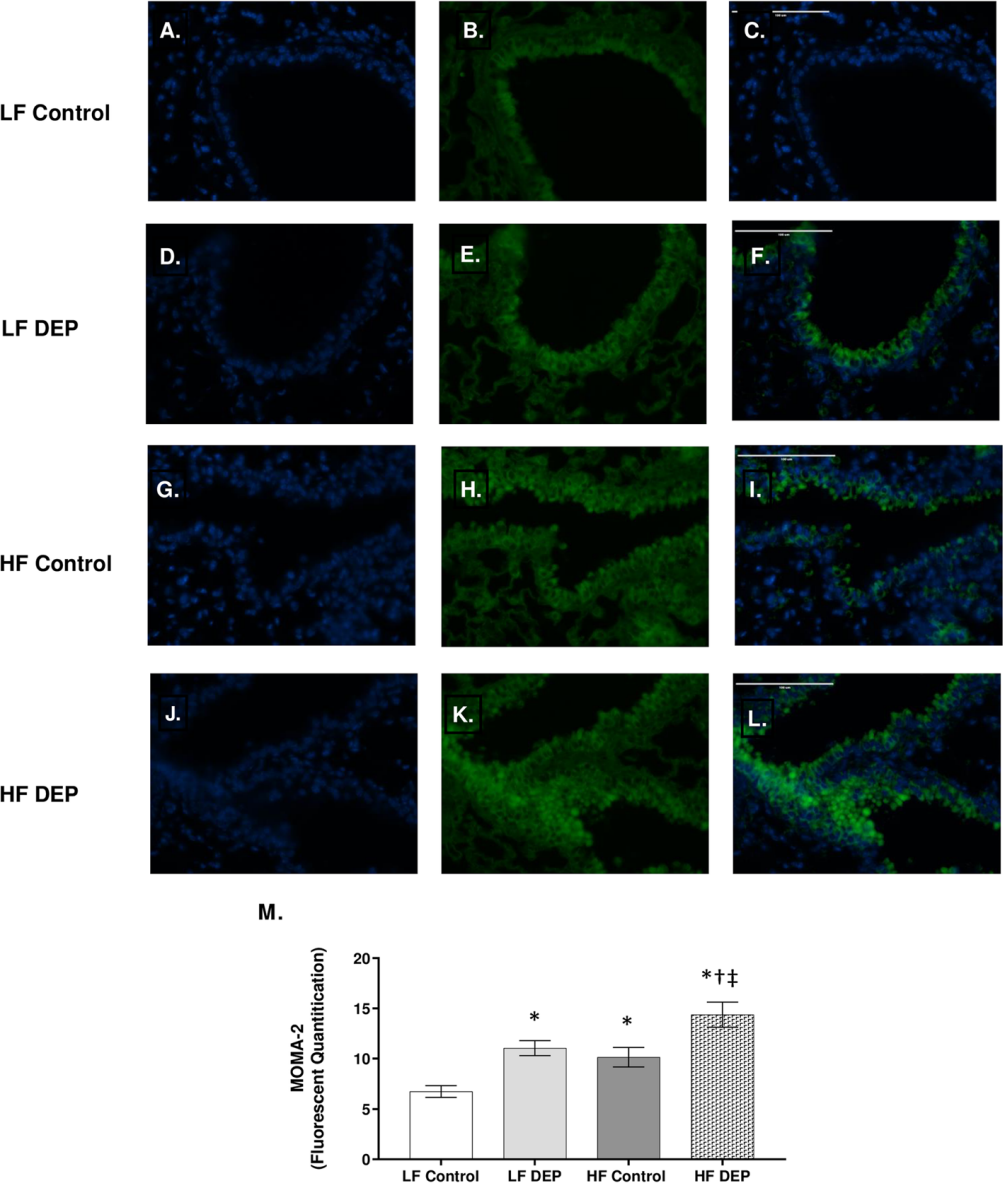
Mice exposed to diesel exhaust particles express higher levels of MOMA-2. Representative images of MOMA-2 expression in the lungs of C57Bl/6 mice on a control (LF; **a-c**) or high-fat (HF; **g-i**) diet exposed to saline (control) or on a LF (**d-f**) or HF diet (**j-l**) exposed to diesel exhaust particles (DEP – 35 μg PM) twice a week for a total of 30 days. Green fluorescence indicates MOMA-2 expression, blue fluorescence is nuclear staining (Hoechst). Right panels (**c**, **f**, **i**, **l**) are merged figures of left (blue; **a**, **d**, **g**, **j**) and center (green; **b**, **e**, **h**, **k**) panels. **m** Graph of histology analysis of lung MOMA-2 fluorescence. 40x magnification; scale bar = 100 μm. Data are depicted as mean ± SEM with *p < 0.05 compared to LF Control, †*p* < 0.05 compared to HF Control, ‡*p* < 0.05 compared to LF DEP by two way ANOVA

Since macrophages are the most abundant steady-state leukocyte, we suspected them of being involved in the observed oxidative stress response [40]. To quantify if the proportion of macrophages were higher in the exposed groups, we stained for monocyte and macrophage antibody-2 (MOMA-2). Compared to LF controls (Fig. 8a-c), we observed that MOMA-2 expression was increased in the LF DEP group (Fig. 8d-f, *p* = 0.002), the HF Control group (Fig. 8g-i, *p* = 0.015), and the HF DEP group (Fig. 8j-l, *p* < 0.001), as quantified in Fig. 8m. The F values for MOMA – 2 are: exposure = 20.13, diet = 12.53, exposure x diet interaction = 0.002.

### Exposure to DEP results in activation of TLR2 and TLR4 in C57Bl/6 mice

**Fig. 9:**
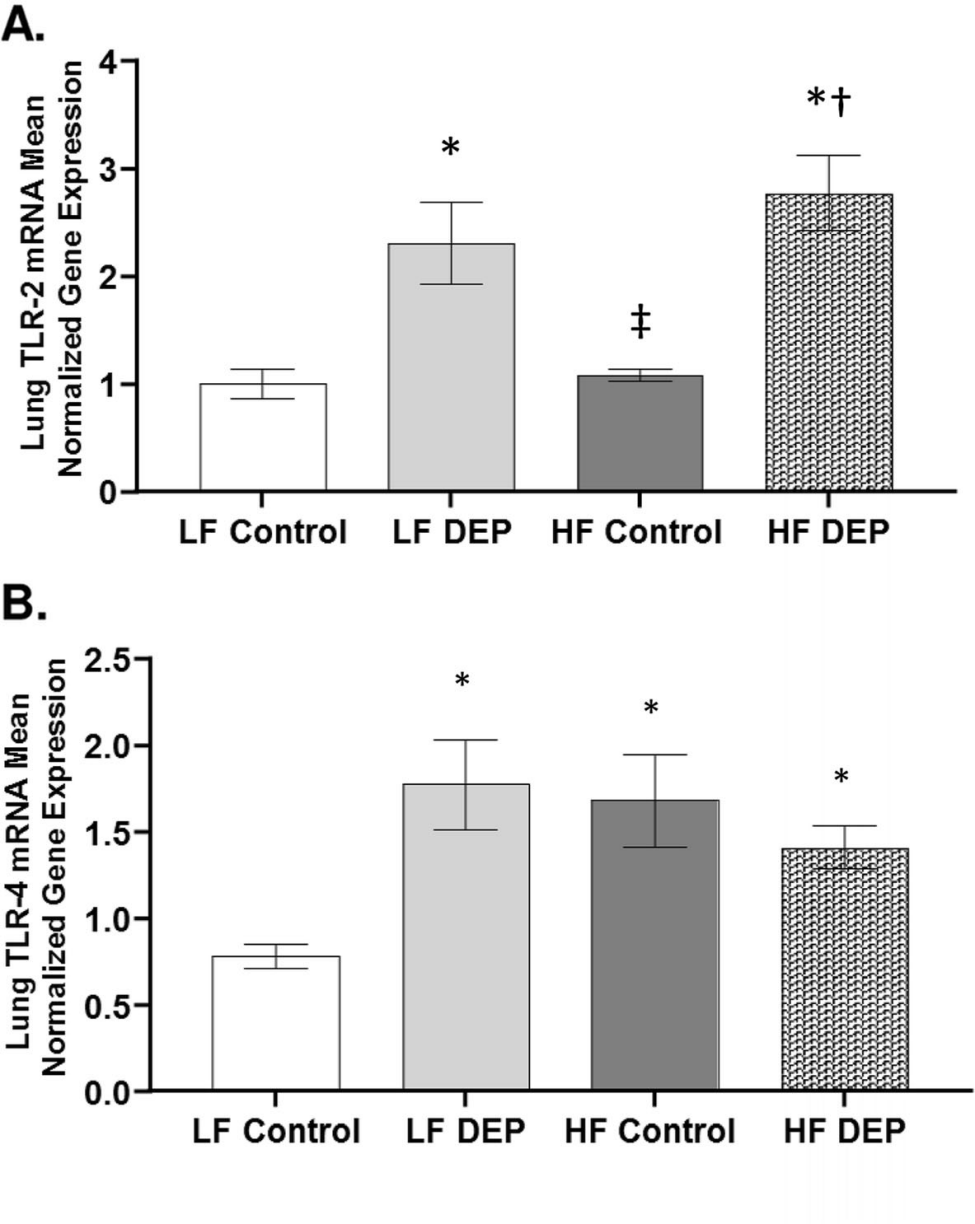
Exposure to diesel exhaust particles results in the activation of TLR2 and TLR4. Mean normalized gene expression of (**a**) TLR2 and (**b**) TLR4 mRNA transcript expression within the lungs of C57Bl/6 wildtype mice, as quantified by RT-qPCR, on either control (LF) or high-fat (HF) diet, exposed to either saline (control) or diesel exhaust particles (DEP − 35 μg PM) twice a week for a total of 30 days. Data are depicted as mean ± SEM with *p < 0.05 compared to LF Control, †*p* < 0.05 compared to HF Control, ‡*p* < 0.05 compared to LF DEP by two way ANOVA

Both resident myeloid and structural cells of the lung express a complete repertoire of TLRs that recognize pathogen-associated molecular patterns (PAMPs) [41]. The TLR family disseminates the signal to intracellular transcription factor - NF-κB that regulates inflammatory gene expression [42]. TLR2 and TLR4 recognize bacterial ligands such as lipoproteins and lipopolysaccharides (LPS), respectively. Since we observed bacterial alterations within the airway lumen, we quantified the expression of TLRs and NF-κB p65 protein. We observed a significant increase in TLR2 regardless of the diet consumed. Compared to LF and HF Controls, we observed a significant increase in the expression of TLR2 mRNA transcript in the lungs of LF DEP (Fig. 9a, *p* = 0.002) and HF DEP (Fig. 9a, *p* < 0.001) exposed animals (Fig. 9a, f = 30.68 for exposure). Compared to the LF Control group, we also observed a statistical increase in TLR4 mRNA in the LF DEP (*p* = 0.004), HF Control (*p* = 0.010), and HF DEP (*p* = 0.050) group (Fig. 9b). The respective F values for TLR4 are: exposure = 3.607, diet = 1.321, exposure x diet interaction = 7.896.

**Fig. 10:**
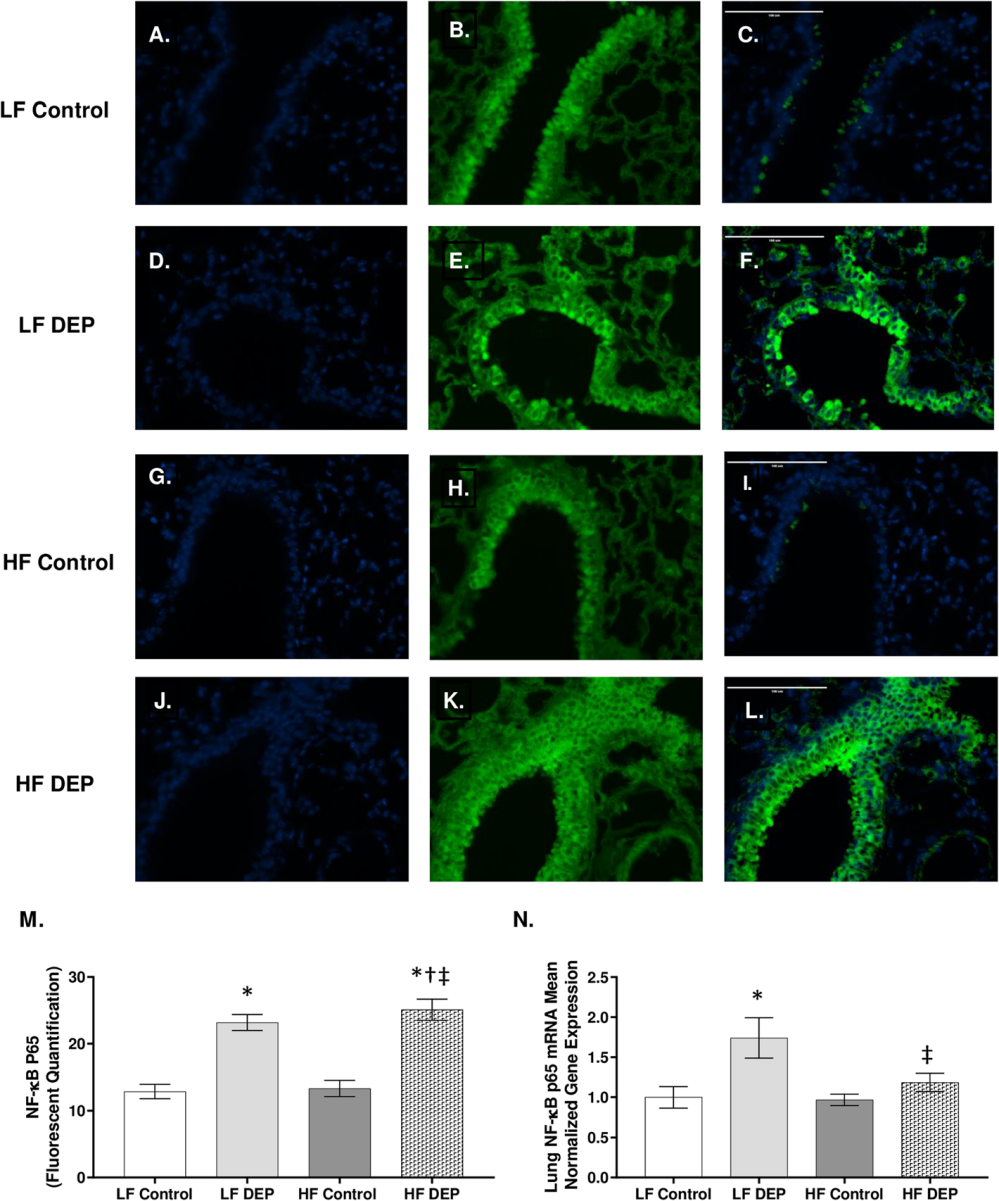
Mice exposed to diesel exhaust particles have increased expression of NF-κB p65. Representative images of NF-κB p65 expression in the lungs of C57Bl/6 mice on a control (LF; **a-c**) or high-fat (HF; **g-i**) diet exposed to saline (control) or on a LF (**d-f**) or HF diet (**j-l**) exposed to diesel exhaust particles (DEP – 35 μg PM/m3) twice a week for a total of 30 days. Green fluorescence indicates NF-κB p65 expression, blue fluorescence is nuclear staining (Hoechst). Right panels (**c**, **f**, **i**, **l**) are merged figures of left (blue; **a**, **d**, **g**, **j**) and center (green; **b**, **e**, **h**, **k**) panels. **m** Graph of histology analysis of lung NF-κB p65 fluorescence. **n** Mean normalized gene expression of NF-κB p65 mRNA transcript expression within the lung, as determined by RT-qPCR. 40x magnification, scale bar = 100 μm. Data are depicted as mean ± SEM with *p < 0.05 compared to LF Control, †*p* < 0.05 compared to HF Control, ‡*p* < 0.05 compared to LF DEP by two way ANOVA

Compared to LF controls (Fig. 10a-c), we observed that NF-κB p65 expression was increased in the LF DEP group (Fig. 10d-f), and the HF DEP group (Fig. 10j-l), as quantified in Fig. 10m (*p* < 0.001, F = 73.89 for exposure). Real-time RT-qPCR of the NF-κB p65 mRNA transcript revealed statistical increases in the LF DEP compared to the LF controls (Fig. 10n, *p* = 0.01; F = 5.705 for exposure). However, we did not observe an increase in NF-κB p65 in the HF DEP group (Fig. 10n).

### C57Bl/6 mice exposed to DEP show increased collagen deposition surrounding the bronchioles

**Fig. 11:**
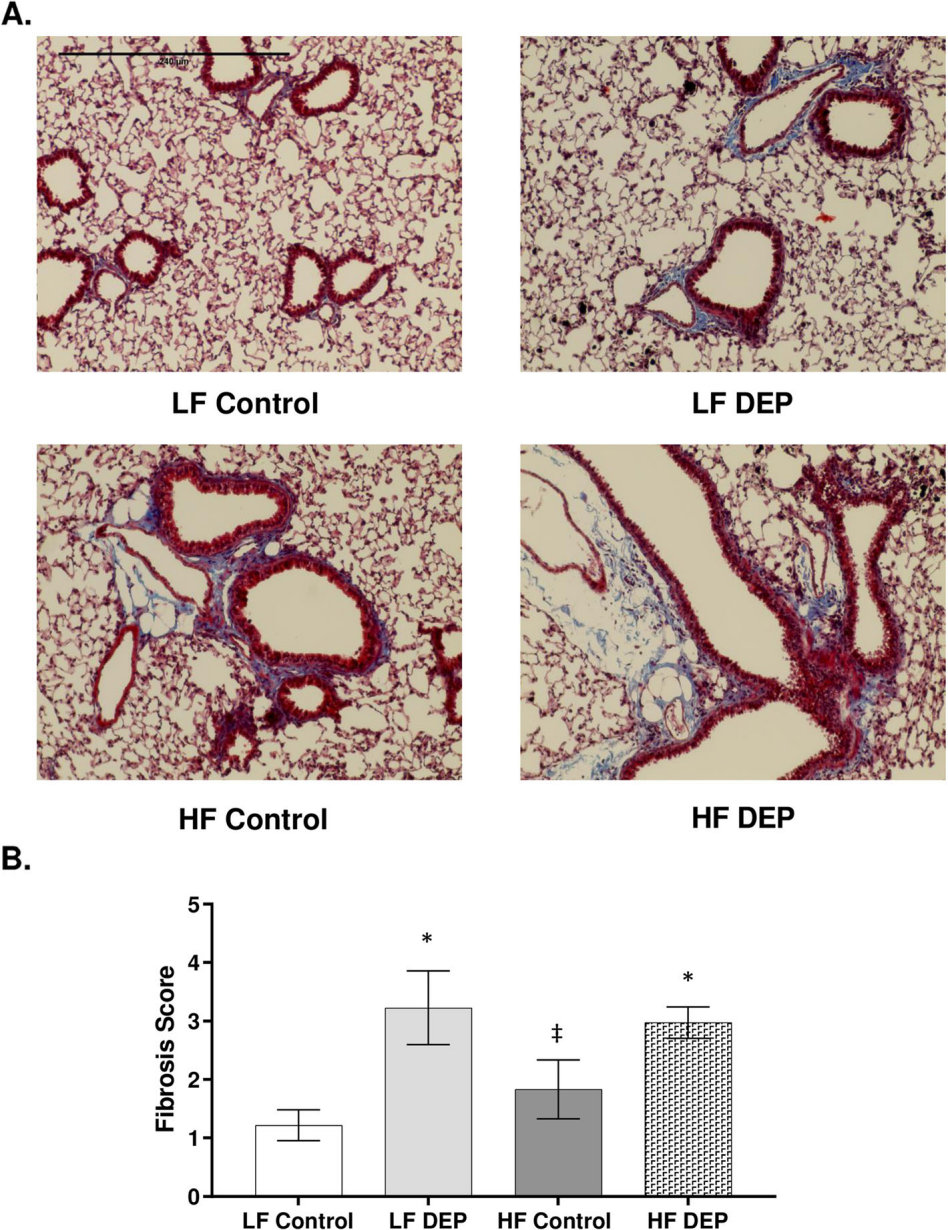
Exposure to diesel exhaust particles results in increased collagen deposition surrounding the bronchioles. **a** Representative images of lung tissue sections stained with Masson’s trichrome in C57Bl/6 wildtype mice on either control (LF) or high-fat (HF) diet exposed to either saline (control) or diesel exhaust particles (DEP – 35 μg PM) twice a week for a total of 30 days. Blue areas indicate collagen deposition. **b** Graph of histology scoring of Masson’s trichrome staining. 20x magnification, scale bar = 240 μm. Data are depicted as mean ± SEM with *p < 0.05 compared to LF Control, ‡*p* < 0.05 compared to LF DEP by two way ANOVA

Air pollutant exposures have been implicated in inducing and/or worsening fibrosis in chronic lung conditions [43, 44]; therefore, we stained for collagen deposition in the lungs following 30 days of subchronic DEP exposure. Compared to LF Controls, we observed increased collagen deposition surrounding the bronchioles in both the LF DEP (Fig. 11b, *p* = 0.002) and HF DEP (Fig. 11b, *p* = 0.007) exposed animals (Fig. 11b, f = 13.12 for exposure), suggesting that subchronic exposures resulted in increased deposition of extracellular matrix components (ECM).

### Probiotic supplementation decreases the expansion of Proteobacteria in C57Bl/6 mice lung

**Fig. 12:**
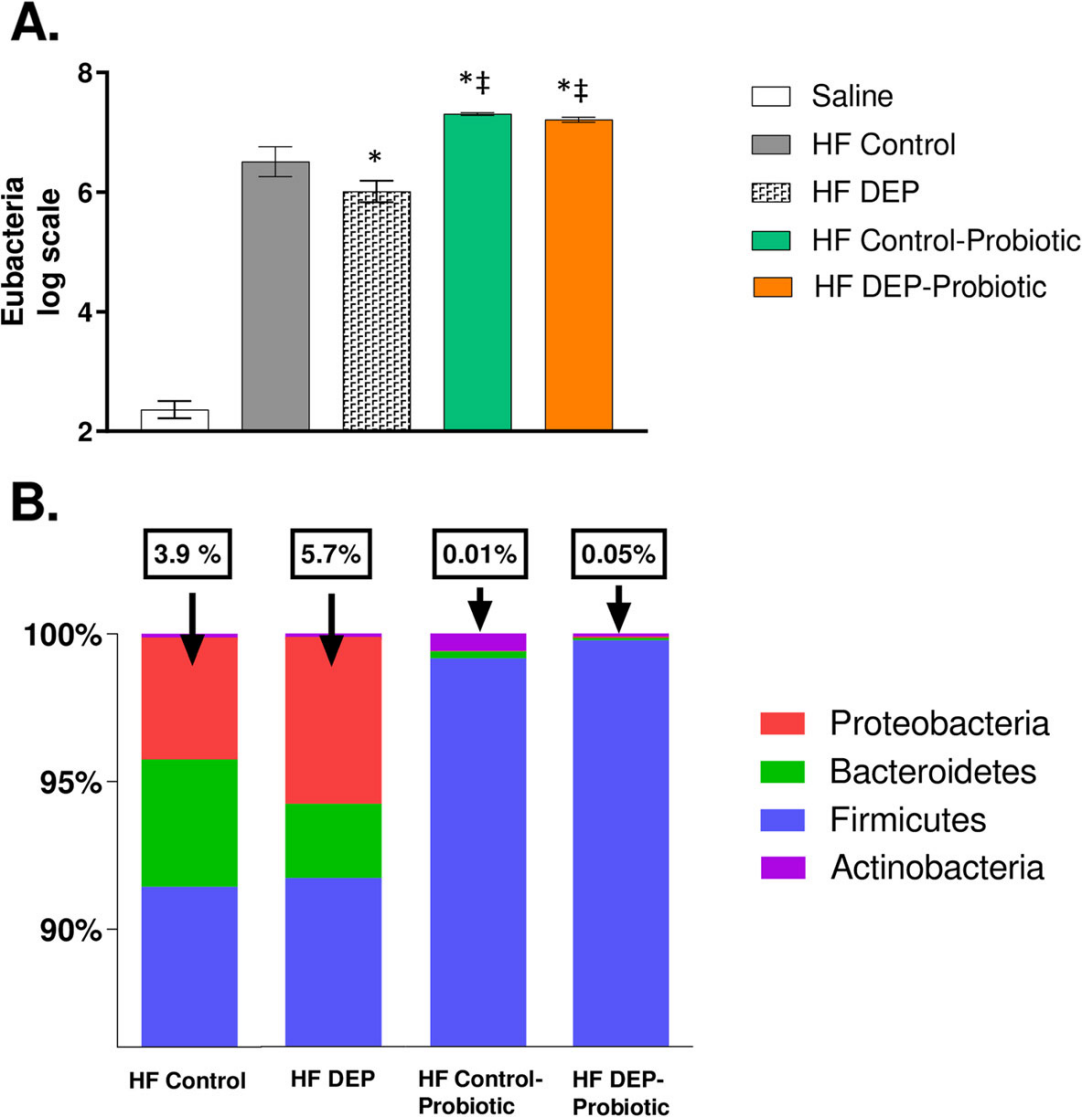
Probiotic supplementation alters the microbial profile and decreases the expansion of Proteobacteria. **a** Quantification by qPCR of (**a**) Eubacteria (log scale), **b** 100% stacked columns representing the percentages of major phyla within the lungs of C57Bl/6 wildtype mice on high-fat (HF) diet exposed to either saline (control) or diesel exhaust particles (DEP – 35 μg PM) twice a week for a total of 30 days alongside a dose of 0.3 g/day (~ 7.5 × 10^7^ cfu/day) of Ecologic® Barrier probiotics in the drinking water over the course of the exposures. Data are depicted as mean ± SEM with *p < 0.05 compared to HF Control, ‡*p* < 0.05 compared to HF DEP by two way ANOVA. The saline group included on the graph is a negative control reference and was not included in the two way ANOVA analysis

Probiotic supplementation has been shown to improve lung immune responses that have beneficial effects during respiratory infections [45]. We investigated whether probiotic intake could aid in restoring the commensal phyla within the lungs. Interestingly, our results revealed an increase in the overall bacterial abundance (Fig. 12a), compared to HF Control (*p* = 0.004) and compared to HF DEP (*P* < 0.001), with a significant increase in Firmicutes and Actinobacteria (Fig. 12b, Supplementary Figure 1) in the probiotic groups. We also observed that the expansion of Proteobacteria was significantly attenuated in the lungs of HF Control and HF DEP-exposed animals compared to their counterparts with no probiotic supplementation (Fig. 12b, Supplementary Figure 1).

### Probiotic supplementation decreases inflammation and collagen deposition

**Fig. 13:**
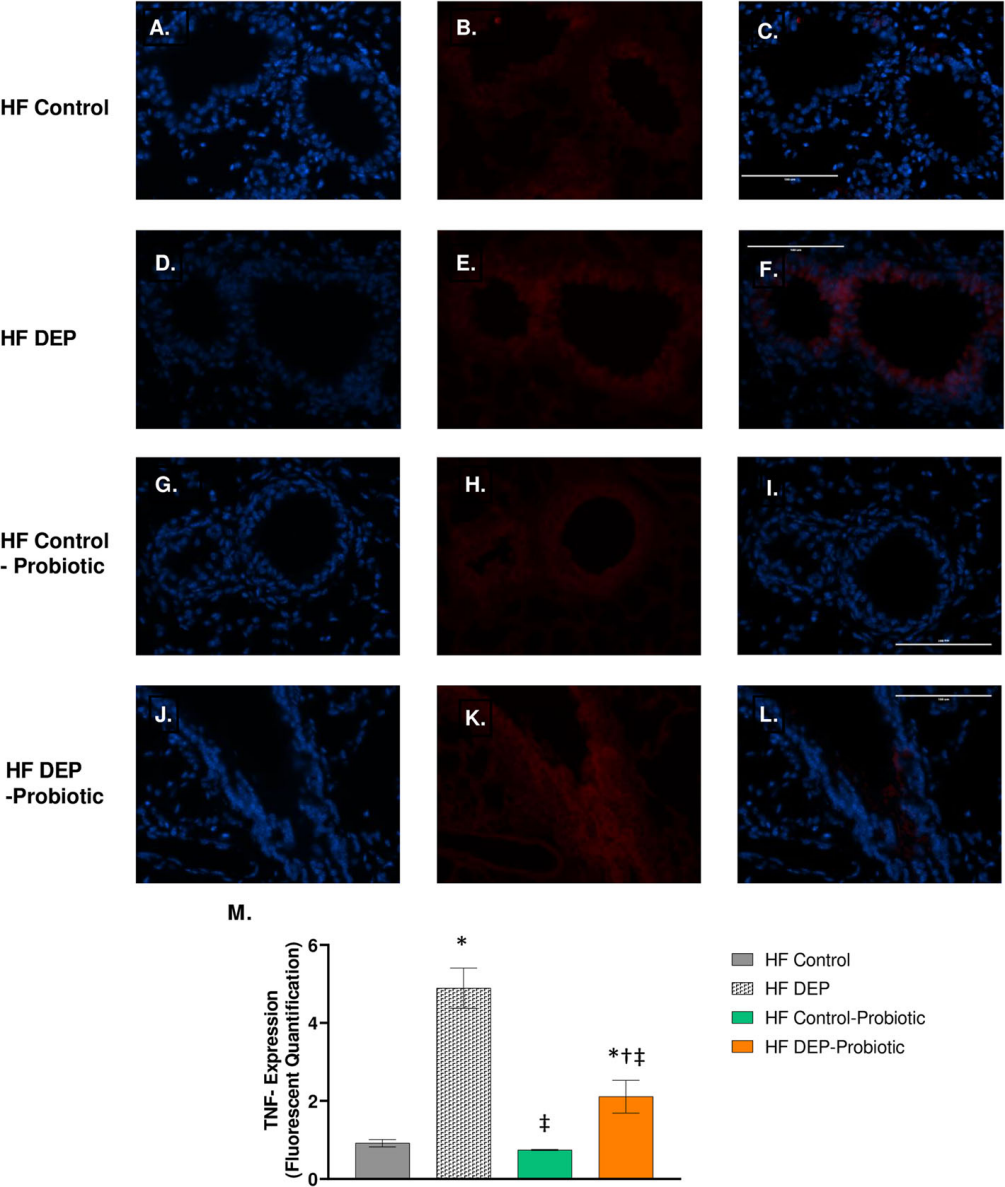
Probiotic supplementation attenuates the pro-inflammatory TNF-α response with diesel exhaust particle exposure. Representative images of TNF-α expression in the lungs of C57Bl/6 mice, on a high-fat (HF) diet exposed to either (**a–c**) saline, (**d–f**) DEP – 35 μg PM, or (**g–i**) saline and probiotics - 0.3 g/day (~ 7.5 × 10^7^ cfu/day) and (**j-l**) DEP and probiotics. Red fluorescence indicates TNF-α expression, blue fluorescence is nuclear staining (Hoechst). Right panels (**c**, **f**, **i**, **l**) are merged figures of left (blue; **a**, **d**, **g**, **j**) and center (red; **b**, **e**, **h**, **k**) panels. **m** Graph of histology analysis of lung TNF-α fluorescence. 40x magnification, scale bar = 100 μm. Data are depicted as mean ± SEM with *p < 0.05 compared to HF Control, †*p* < 0.05 compared to HF Control - Probiotics, ‡*p* < 0.05 compared to HF DEP by two way ANOVA

Probiotics have been shown to improve inflammation observed in many lung diseases due to their immunomodulatory properties [46–48]. Thus, we investigated whether the inflammation observed with DEP could be attenuated with probiotics. We observed that TNF-α expression, found to be significantly elevated with DEP exposures, decreased with probiotic supplementation. In comparison to the lungs from the HF DEP (Fig. 13d-f), we observed a significant decrease in TNF-α in the lungs from the HF Control – Probiotics (Fig. 13g-i) and HF DEP – Probiotics group (Fig. 13j-l, *p* < 0.001, F = 63.22 for exposure). Although the decrease in TNF-α expression is significant, we notice that the expression in the HF DEP – Probiotics group is still significantly higher when compared to HF Control (Fig. 13a-c, *p* = 0.015) and HF Control – Probiotics group (Fig. 13 G-I, *p* = 0.005). The respective F values for TNF-α are: exposure = 63.22, probiotic treatment = 19.45, exposure x probiotic interaction = 15.11.

**Fig. 14:**
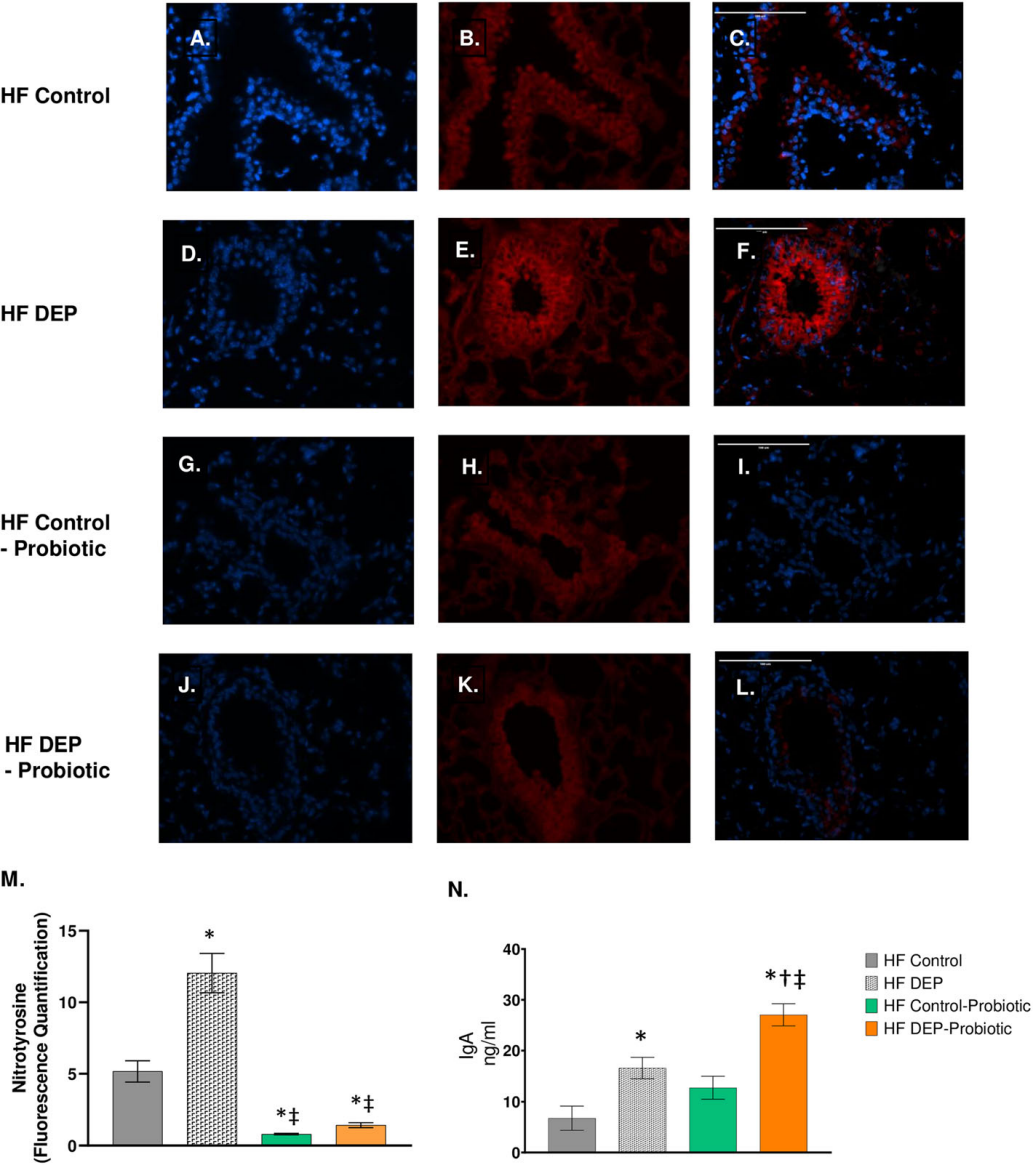
Probiotic supplementation decreases nitrotyrosine expression and enhances IgA production. Representative images of nitrotyrosine expression in the lungs of C57Bl/6 mice, on a high-fat (HF) diet exposed to either (**a–c**) saline, (**d–f**) DEP – 35 μg PM, or (**g–i**) saline and probiotics - 0.3 g/day (~ 7.5 × 10^7^ cfu/day) and (**j-l**) DEP and probiotics. Red fluorescence indicates nitrotyrosine expression, blue fluorescence is nuclear staining (Hoechst). Right panels (**c**, **f**, **i**, **l**) are merged figures of left (blue; **a**, **d**, **g**, **j**) and center (red; **b**, **e**, **h**, **k**) panels. **m** Graph of histology analysis of lung nitrotyrosine fluorescence. **n** Quantification of IgA (ng/ml) in the bronchoalveolar lavage fluid by ELISA. Data are depicted as mean ± SEM with *p < 0.05 compared to HF Control, †*p* < 0.05 compared to HF Control - Probiotics, ‡*p* < 0.05 compared to HF DEP by two way ANOVA

We observed a similar statistical decrease in nitrotyrosine in the HF Control – Probiotic (Fig. 14g-i) and HF DEP – Probiotic group (Fig. 14j-i) compared to the HF DEP (Fig. 14d-f, *p* < 0.001) and HF Control (Fig. 14a-c, *p* < 0.01) (Fig. 14d-f). The F values for nitrotyrosine are: exposure = 22.81, probiotic treatment = 90.74, exposure x probiotic interaction = 15.86. When we checked to see if immunoglobulin levels were altered with probiotic treatments (Fig. 14n), we observed that IgA was significantly elevated in the HF DEP – Probiotics group when compared to the HF Control (*p* < 0.001), HF DEP (*p* = 0.001) and HF Control – Probiotics (*p* < 0.001) group. The F values for IgA levels are: exposure = 28.74, probiotic treatment = 13.41, exposure x probiotic interaction = 1.016.

**Fig. 15:**
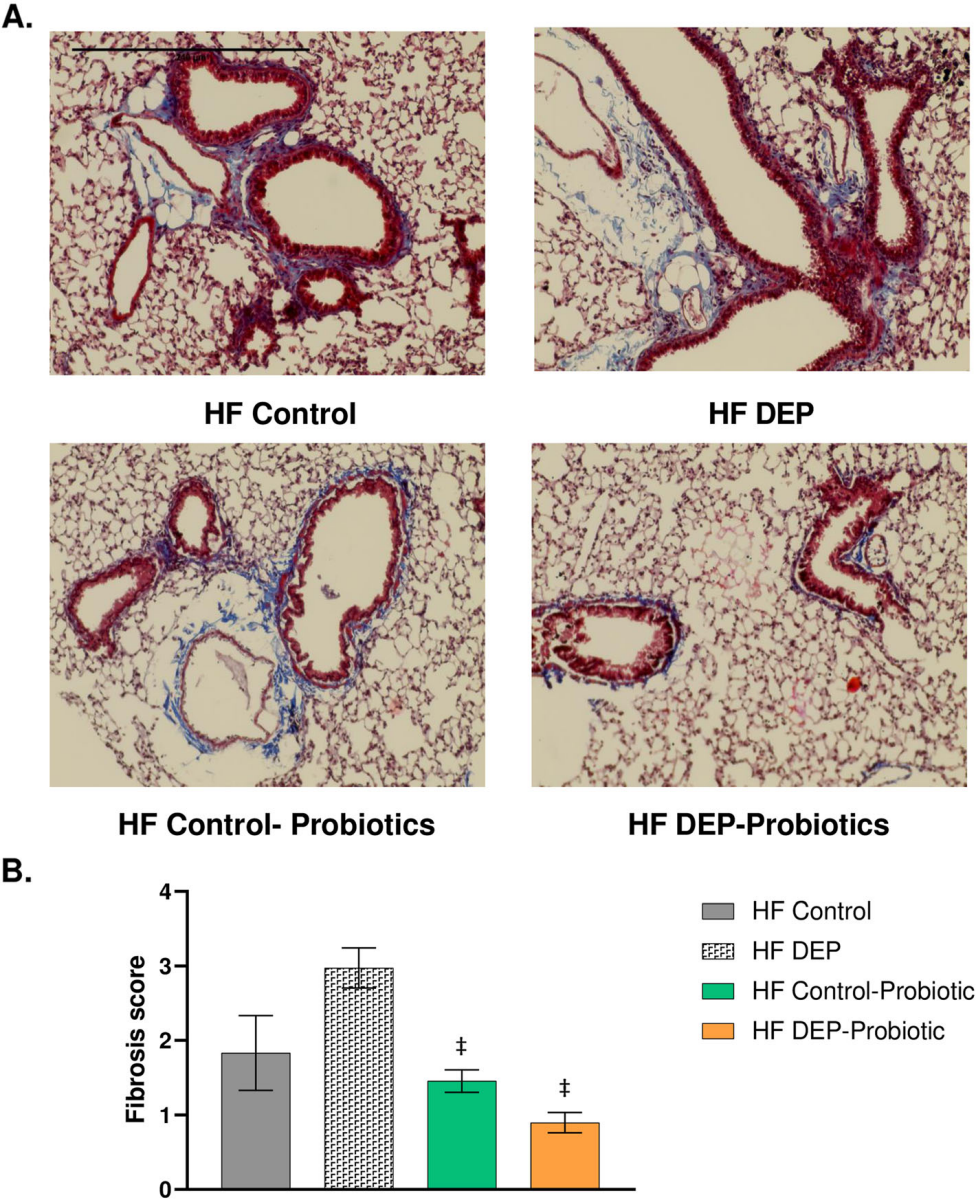
Probiotic supplementation decreases collagen deposition surrounding the bronchioles. **a** Representative images of lung tissue sections stained with Masson’s trichrome in of 4–6 week-old C57Bl/6 wildtype mice on high-fat (HF) diet exposed to either saline (control) or diesel exhaust particles (DEP – 35 μg PM) twice a week for a total of 30 days alongside a dose of 0.3 g/day (~ 7.5 × 10^7^ cfu/day) of Ecologic® Barrier probiotics in the drinking water over the course of the exposures. Blue areas indicate collagen deposition. **b** Graph of histology scoring of Masson’s trichrome staining. 20x magnification, scale bar = 240 μm. Data are depicted as mean ± SEM with ‡*p* < 0.05 compared to HF DEP by two way ANOVA

With the observed decrease in inflammation with probiotic supplementation, we investigated whether the pathological outcomes observed with DEP exposure could be attenuated. Interestingly, we observed that collagen deposition was significantly decreased in the HF DEP – Probiotics group and HF Control – Probiotics group when compared to the HF DEP group (Fig. 15b, *p* = 0.002). The F values for fibrosis score are: exposure = 2.191, probiotic treatment = 11.24, exposure x probiotic interaction = 1.821.

## Discussion

Recent studies have demonstrated a strong correlation between exposure to air pollutants, including PM, and increased incidence and hospitalizations of respiratory conditions such as asthma and COPD. Lung diseases are often associated with persistent colonization of bacteria belonging to the Proteobacteria phylum [49, 50]. Although alterations in the commensal lung microbiome are postulated to influence disease behaviors, a direct association between exposure to particulate air pollution components and bacterial lung microbial alterations have not been characterized. Additionally, while HF diet consumption is understood to increase inflammation and cause gut microbial shifts, little information exists on the combined effects of HF diet and PM pollution within the lungs. In this study, we report for the first time to our knowledge that exposure to DEP alters the microbiome and inflammatory response within the lungs of C57Bl/6 mice on both LF and HF diets.

We observe a pronounced increase in neutrophil counts in the HF DEP groups and the HF Control group, which reveals an interaction between diet and exposure, suggesting that the synergistic effects exacerbate the observed acute immune response. Although we observe an increase in monocytes and lymphocytes, the involvement of neutrophils is far more pronounced. Neutrophils are among the first cells to be recruited to the site of infection, and it is likely they play a crucial role in mediating acute immune responses with DEP exposures [51]. Few studies point to the elevation of several pro-inflammatory cytokines, including TNF-α, IL-1β, IL-6, and IL-8, after acute DEP exposures [52–54]. However, in our study, except for TNF-α most of these initial pro-inflammatory cytokines were not elevated, likely due to the longer study duration. We did, however, observe a significant increase in IL-10 in the lungs of DEP-exposed animals, regardless of the diet they were consuming. Thus, it is plausible that at the 30 day exposure time point, there may be activated anti-inflammatory mediators. We suspect these inflammatory cytokines are generated predominantly by macrophages that were found to be abundant in the DEP exposed groups. Based on our results, as well as previous studies, it is likely that there is macrophage polarization into pro-inflammatory M1 producing TNF-α and anti-inflammatory M2 producing IL-10 occurring within the lungs after 30 days of DEP exposure [55].

Acute exposure to DEP has been shown to upregulate Muc5ac and Muc5b [56], but we were unable to detect these mucins at the 30 day exposure time point. However, we were able to detect increased overall mucus in the exposed groups by AB/PAS staining within the lungs, which suggests that DEP exposure results in the sustained presence of mucus after 30 days of exposure. The presence of excessive mucus has been shown to cause the expansion of the opportunistic pathogen, *Pseudomonas aeruginosa* belonging to the Proteobacteria phylum, which is commonly observed in lung diseases [57]. In addition to mucus, the DEP-induced inflammation also likely changes the nutrient environment within the lungs. Although inflammation was traditionally understood to function as a means to expel potential antigens, it is now understood that inflammatory by-products, such as ROS and RNS, result in the generation of nitrates that can be utilized only by bacteria within the Proteobacteria phylum [58]. Proteobacteria can efficiently metabolize nitrates since they have the highest nitrate reductase activity in their genomes, compared to the other commensals [38]. We observed a significant elevation in peroxynitrite levels in the lungs of our DEP-exposed animals that was correlated with an increased abundance of Proteobacteria in the lung microbiome, which suggests that extracellular nitrate availability is aiding the proliferation of Proteobacteria. Firmicutes and Bacteroidetes do not possess nitrate-reducing metabolic capabilities, which we speculate may drive the decreased abundance in the HF groups. ROS-RNS are generated by many immune cells, including neutrophils and macrophages, as anti-microbial effectors [59]. Although we see an increase in systemic neutrophils with acute exposures, it is likely most of the ROS-RNS produced is generated by macrophages since they are the most abundant within the lungs and have a longer life-span when compared to neutrophils [60]. We noticed that the bacterial abundance was higher in the BALF when compared to the lung tissues, suggesting that most of the lung bacteria are present within the airway lumen (data not shown).

Endotoxins present on PM have been shown to upregulate TLR2, which signals the pIgR to facilitate increased IgA transport across the airway lumen [61]. Since we observe an increase in pIgR in the DEP exposed groups, it is plausible that the increase in IgA is in response to a TLR mediated signaling [62]. The increase in TLR4 observed in the HF Control group is possibly due to the demonstrated ability of TLR4 signaling in the obesity-induced inflammation [63]. Although we do not observe an increase in IgA, IgG is elevated in the HF Control groups. This may be due to a gut-lung mediated response since increased IgG has been shown with HF diet consumption [64, 65]. TLRs have also been shown to activate macrophages with the subsequent transcription of inflammatory mediators like TNF-α [66]. All TLRs converge into activating the NF-κB transcription factor family, and NF-κB signaling is understood to be required for sustained cytokine expression in macrophages [67]. Thus, the increased expression of the NF-κB protein p65 observed within the lungs in response to DEP could be due to TLR-mediated signaling; however, additional studies are necessary to determine the mechanistic signaling pathways involved.

**Fig. 16:**
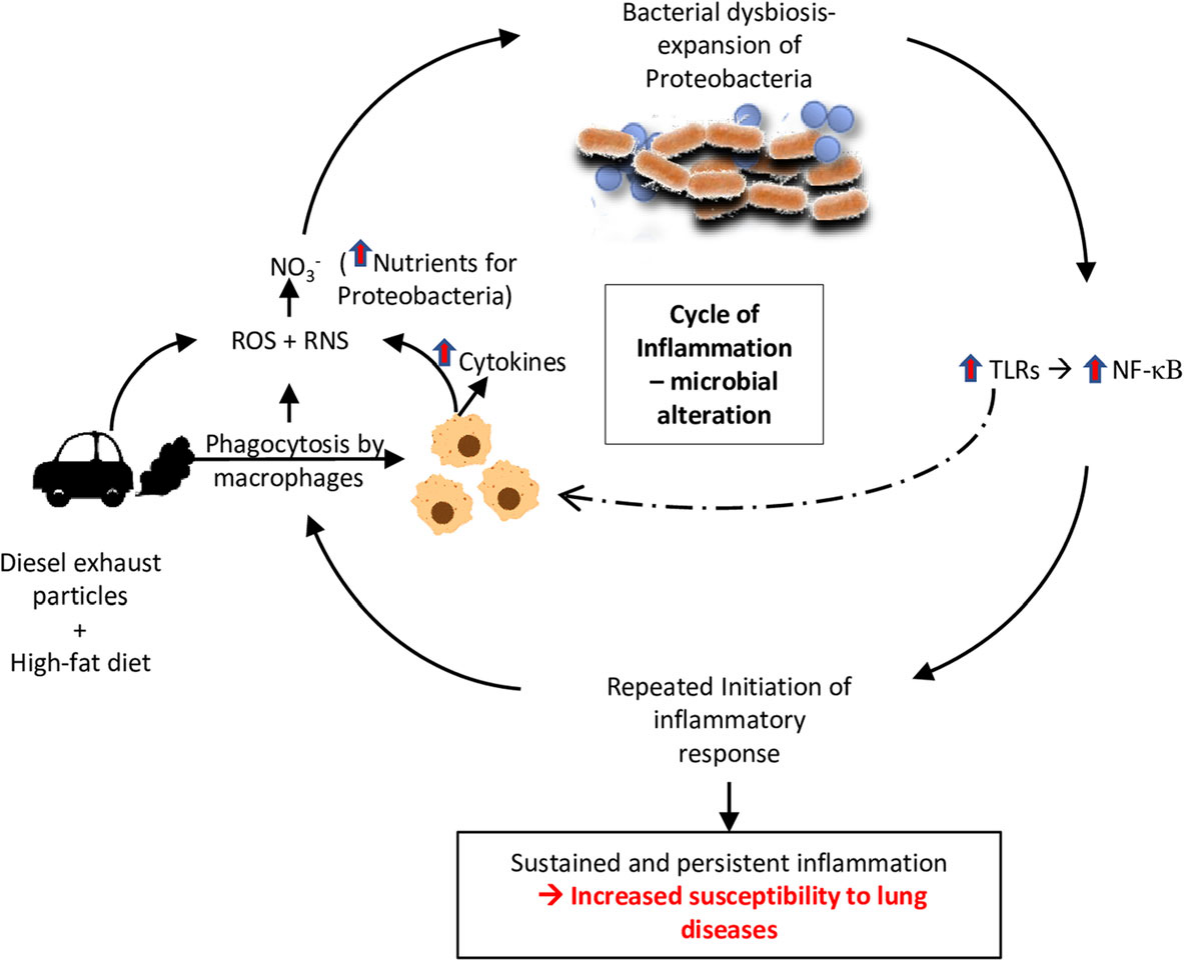
Synergistic effects of diesel exhaust particles (DEP) and high-fat (HF) diet results in increased reactive oxygen and nitrogen species (ROS + RNS) produced possibly by macrophages, which increases nitrates within the lung environment. These nitrates provide nutrients for anaerobic respiration and selective growth of Proteobacteria within the lungs resulting in alterations in the commensal microbial composition. Both DEP and the bacterial alterations could activate Toll-like receptors (TLRs) which results in the subsequent activation of NF-κB mediated inflammatory gene transcription. Since NF-κB signaling is understood to be required for sustained cytokine expression in macrophages, the production of inflammatory cytokines, ROS, and RNS is sustained, resulting in a continuous cycle of inflammation and microbial shifts throughout the duration of DEP exposures. This sustained and persistent inflammation is a possible contributor and significant in the development of lung diseases

TLR4 is activated in response to the gram-negative bacterial cell wall component - LPS [68]. The expansion of gram-negative Proteobacteria observed within the airway lumen could contribute to the activation of TLR4. There is likely a continuous cycle of inflammation throughout the duration of DEP exposures – first by DEP-induced inflammation, which increases nitrates within the lung environment selectively, enabling the outgrowth of Proteobacteria. Secondly, the expansion of Proteobacteria could contribute to TLR4 activation, making this a vicious cycle (Fig. 16). Although it is possible that endotoxin on the DEP material could also result in the activation of TLR4, our data show that there is negligible amounts of endotoxin on the DEP material (Supplementary Figure 2). This observed response is analogous to the inflammation-dysbiosis model proposed in the exacerbation of chronic lung diseases [69]. Our data suggest that in otherwise healthy individuals with no underlying pulmonary diseases, exposure to PM pollutants can increase the susceptibility to develop pathological lung conditions since we observed increased college deposition surrounding the bronchioles with 30 days of DEP exposure in wildtype C57Bl/6 mice. Chronic inflammation resulting from immune responses that persist for longer durations in which inflammation, tissue remodeling, and repair processes coincide has been shown to induce fibrosis [70]. Oxidative stress has been demonstrated to contribute to the progression of IPF and other lung diseases [71]. We noticed abnormal tissue elements in our morphological assessments, identified to be collagen deposition by Masson’s trichrome staining. This suggests that there is sustained and persistent inflammation resulting in the deposition of ECM. Although the initial inflammatory response may facilitate the removal of the DEP particles, repeated exposure of these particles within the lungs leads to enhanced inflammation and pathological outcomes. The increase in ECM components is an observed phenomenon in lung diseases such as IPF and COPD [72, 73]. Interestingly, both these diseases show persistent colonization of bacteria such as *Haemophilus influenzae* and *Pseudomonas aeruginosa* both of which belong to the Proteobacteria phylum [74]. We suspect that alterations in the commensal lung microbiome in individuals with no prior lung conditions can increase inflammation and facilitate the proliferation of pathogenic bacteria if not mediated by host immune responses.

Interestingly, and somewhat unexpectedly, with probiotic supplementation, we observe that the expansion of Proteobacteria is curbed with a decrease in nitrates in the lung environment, which indicates that the DEP-induced inflammation is attenuated. The mechanism by which probiotics help attenuate inflammation has been reported to involve T_reg_ cell-mediated balance of pro-inflammatory signaling [42, 67]. Thus, it is plausible that pro-inflammatory responses are balanced within the DEP or HF lungs with probiotics, as we observe a reduction in expression of the pro-inflammatory cytokine TNF-α. The reduced pro-inflammatory and oxidative stress responses may play a role in decreasing collagen deposition. Since the effects of probiotics are primarily thought to be via a gut-lung mediated response, further studies investigating how these probiotics modulate inflammatory responses within the gastrointestinal tract are currently being pursued by our lab. Although we suspect the pathogenic bacteria are eliminated (or “out-competed”) in this process, it is important to consider whether a shift in the commensal microbiota is ideal. Proteobacteria have been shown to induce IgA responses in the gastrointestinal tract during the first week of life [75]. Proteobacteria’s healthy balance within the commensal microbial profile has also been shown to protect against sepsis [76]. However, to our knowledge, there is no evidence of Proteobacteria inducing immune responses within the adult lungs. Despite this, we checked to see if there were alterations in IgA with probiotic exposures. We observed an increase in IgA with probiotic intake, which is in cooperation with other studies suggesting that immune defenses may not be affected by the elimination of Proteobacteria from the lungs [77]. However, further studies are warranted to determine if susceptibility to bacterial or viral challenges is influenced by probiotic supplementation. Since only the expansion of Proteobacteria is concerning, rather than causing a shift from the commensal profile, strategies to reduce Proteobacteria selectively could be explored. Eliminating nitrates within the lungs through the use of antioxidants to scavenge radicals or inhibiting molybdenum-dependent enzymes by Proteobacteria necessary for nitrate utilization are viable alternatives for further characterization [78].

Although we observe an alteration within the lung microbes at a 30-day time point, these responses may occur in phases. Our observations at a certain time point within a group may not necessarily be identical to another time point, especially considering the lung microbes are highly variable. These exposures were done for a total of 30 days and the reported findings are taken from only the one-time point, which is a limitation to this study. However, it is important to consider that these exposures were done twice a week and not daily, which allowed for some clearance and immune mediation of these particles within the lungs. Also, exposures done twice a week as opposed to daily exposures are similar in nature to human exposures on days of high vs. acceptable PM levels. The concentration used in the current study represents a dose of ~ 10 μg/mouse per day, as previously described, which is estimated to be approximately 40-fold higher than the comparable alveolar deposition from a 24-h inhalation of 100 μg/m^3^ in man when adjusted for mice [79]. This is assuming that all the DEP reaches the alveoli; however, it plausible that there may be at least some clearance of the DEP via the mucociliary escalator. It is also important to note that many urban regions worldwide exceed 100 μg/m^3^ daily PM_2.5_ levels [80]. While the dose chosen for the current study may be higher than that experienced by typical human environmental exposures, the (inflammatory) findings in the lung are in agreement with previous DEP-exposure studies [79, 81, 82]. Furthermore, the use of this DEP material is not necessarily representative of all traffic- or industry-generated PM, by current engine standards. This particular DEP material was chosen for the current study because it has been well characterized and previously documented to initiate pulmonary inflammation using the same dose and exposure protocols [79]. The previous characterization allowed the current investigation to determine whether DEP exposure, associated with pulmonary inflammation, promoted alterations in lung microbiota profiles and whether these detrimental outcomes could be mitigated through probiotic-treatment. As such, this serves as a foundational study for the effects of vehicle exhaust-generated DEP on lung microbiota profiles and associated signaling pathways that may contribute to increased susceptibility of pulmonary diseases.

## Conclusions

Our observations within the lungs lead us to hypothesize that DEP exposures cause inflammation and microbial alterations in a ROS-RNS mediated fashion, exacerbated by concurrent consumption of an HF diet. The DEP exposures contributed to a cycle of inflammation and microbial alterations, which could be associated with increased susceptibility to developing fibrosis and associated decrease in lung function with long-term exposures. According to WHO, air pollution exposures alone result in around 7 million premature deaths in a year, of which 43% are deaths due to COPD, and 26% due to respiratory infection deaths [83]. There are close to 380 million people living with COPD, and it is expected to become the leading cause of death in 15 years [84]. Our study suggests that understanding the lung microbiota shifts in response to PM and diet is paramount to identifying the mechanistic pathways involved in air-pollutant mediated effects on lung diseases and overall health.

## Materials and methods

### Animals and inhalational exposure

Four-to-six week-old male C57Bl/6 mice from Taconic, C57BL/NTac were placed on either a control (LF) diet with matched sucrose levels containing 10% fat, 70% carbohydrate and 20% protein (Research Diets # D124505H, *n* = 24) or a high-fat (HF) diet containing 45% fat, 35% carbohydrates and 20% protein (Research Diets #D12451, *n* = 24). A separate set of mice (*n* = 24) were placed on the HF diet and treated with a dose of 0.3 g/day (~ 7.5 × 10^8^ cfu/day) of Ecologic® Barrier probiotics (Winclove Probiotics, Amsterdam, Netherlands) in the drinking water over the course of the exposures. The probiotics used includes a blend of the following bacterial strains: Bifidobacterium bifidum W23, Bifidobacterium lactis W51, Bifidobacterium lactis W52, Lactobacillus acidophilus W37, Lactobacillus brevis W63, Lactobacillus casei W56, Lactobacillus salivarius W24, Lactococcus lactis W19 and, Lactococcus lactis W58. The strains were blended in a carrier matrix of maize starch and maltodextrins with a viable cell count of 2.5 × 10^9^ CFU/gram. Probiotics were changed daily between 4:00–6:00 pm in sterilized bottles to ensure no bacterial growth. Administration in drinking water was chosen over oral gavage to minimize handling and reduce stress for mice [85]. Doses of probiotic were monitored through the use of low-drip metered water bottles, and concentrations adjusted as necessary, to maintain consistent dosing throughout the exposure period. Probiotic approximate dosages were determined on daily consumption of probiotic and water mixture per cage divided by number of mice per cage according to ref. [86]. We had a total of 22 cages, 4 male mice per cage in all groups except animals allotted for histological assessments were grouped 3 per cage. Mice were randomly assigned to be exposed via oropharyngeal aspiration (OA) to 35 μg diesel exhaust particles (DEP, *n* = 36), obtained from NIST (Standard Reference Material #2975), suspended in 35 μl 0.9% sterile saline (VEDCO NDC 50989–641-17) or sterile saline only (control, *n* = 36) twice a week for 30 days, representing a dose of approximately 10 μg/mouse per day, as previously described [79]. At this concentration, the mean particle size of DEP was reported to be 257 ± 46 nm, and resulted in lung, but not systemic, inflammation in C57Bl/6 mice [79]. For OA, mice were lightly anesthetized with 2% Isoflurane (Butler Schein Animal Health) and suspended at an approximately 60-degree angle on a surgery board (rodent intubation stand; Biolite RIS 100). Thirty-five microliter of either DEP suspended in sterile saline, or sterile saline only (vehicle), was pipetted into the oropharynx with a micropipette while the tongue was gently pulled forward with forceps to open the airway. The nostrils were then covered gently to induce aspiration into the lungs. Mice were monitored for any signs of distress during recovery from anesthesia. Mice were housed in standard shoebox caging and maintained at a constant temperature and humidity. Mice had access to chow and water ad libitum throughout the study period. All procedures were approved by the University of North Texas Institutional Animal Care and Use Committee (IACUC) and adhere to the Guide for the Care and Use of Laboratory Animals published by the US National Institutes of Health (NIH Publication No. 85–23, revised 1996).

The concentration for DEP exposure chosen was higher than what would be expected for daily environmental human exposure (see Discussion section). This specific concentration for DEP exposure was chosen because it is similar to the concentration of total PM used in preliminary analysis containing mixtures of whole vehicle exhaust (PM + gases) [87]. OA was chosen as the preferred route for exposure for these studies since it stimulates the route PM would take from the oropharynx region into the lungs and ensures homogenous delivery to the respiratory system.

At the end of the study, mice were anesthetized with Euthasol® and euthanized by exsanguination within 24 h of the final exposure. The lungs were collected from all study animals, snap-frozen, and stored at − 80 °C for RNA analysis. Lung tissues were immediately harvested and prepared for histochemical, immunofluorescent, and protein examination of proposed endpoints. Bronchoalveolar lavage fluid (BALF) was collected by flushing the trachea once with 2 ml of sterile saline. BALF was centrifuged at 200 g for 5 min at 4 °C and the supernatant (1 ml) was collected and stored at − 80 °C for protein analysis. For microbiome analysis, BALF was again centrifuged at 15,000 *g* for 15 min at 4 °C and the pellet was stored at − 80 °C.

The nomenclature used are as follows: (a) LF Control: C57Bl/6 mice placed on LF diet and given sterile saline, (b) LF DEP: C57Bl/6 mice placed on LF diet and exposed to DEP, (c) HF Control: C57Bl/6 mice placed on HF diet and given sterile saline, (d) HF DEP: C57Bl/6 mice placed on HF diet and exposed to DEP, (e) HF Control – Probiotics: C57Bl/6 mice placed on HF diet, treated with probiotics and given sterile saline, (f) HF DEP – Probiotics: C57Bl/6 mice placed on HF diet, treated with probiotics and exposed to DEP.

### Blood differential count

Tail vein blood was collected (*n* = 5 per group) on day five after DEP exposures for a differential count. The blood sample was smeared on a microscopic slide and air-dried following fixation in methanol. Giemsa staining was done using automated slide Stainer (Hematek 3000 Siemens). Blood differential counts were performed as described in ref. [88].

### Histology

For all histological endpoints in lung tissues, the whole lungs were fixed in 10% neutral buffered formalin, embedded in paraffin, and sectioned at 5 μm. Tissues were cleared with HistoChoice Clearing Agent three times for 10 min at RT, rehydrated, and stained. A minimum of 3–4 locations on each section (4 sections per slide), 3 slides, and *n* = 2–3 per group were used for analysis; All histology sections were imaged with bright field illumination at 20x.

#### Alcian blue/PAS staining

Overall mucus production was analyzed by staining with a combination of Alcian-blue/periodic acid-Schiff (AB/PAS) stain as per manufacturer protocols (Fisher Scientific #88043, #88016). The combination of Alcian blue and the PAS stain was used to quantify both neutral (magenta) and acidic (deep blue) mucins. Cells containing both neutral and acidic mucins stained purple. These slides were then scored by a blinded participant on a 15 point scale. The intensity of the colors magenta, deep blue, and purple were each quantified on a scale of 1–5 and the scores were added resulting in a total scoring range of 0 to 15.

#### H&E staining

Morphological analysis of lung tissue sections were done by H & E staining (American Mastertech stain kits, #KTHNEPT), following manufacturer recommendations. Histological changes were analyzed by a blinded participant using a 4-point scale as follows: No detectable inflammation - 0, bronchioles surrounded by a few inflammatory cells – 1, bronchioles surrounded by a layer one cell deep – 2, bronchioles surrounded by a layer 2–4 cells deep – 3, bronchioles surrounded by a layer more than four cells deep – 4.

#### Masson’s trichrome staining

Collagen deposition was analyzed by Masson’s trichrome staining, following manufacturer recommendations (Fisher Scientific, #87019). Scoring was performed by using a Cytation5 imaging reader (BioTek), Gen5 software. For Cytation scoring, each tissue section was divided into six quadrants and each quadrant was imaged as a 2 × 2 montage at 20x in color bright field mode. Images were analyzed, in the red channel which allows for all fibrotic tissue (blue) to be subtracted from the image resulting in the percentage confluence for the red channel. The percentage of fibrosis was calculated by subtracting red percentage confluence from 100.

### Immunofluorescence analysis of the lungs

Immunofluorescent staining was used to quantify TNF-α, IL-10, nitrotyrosine, MOMA-2, and NF-κB p65 proteins using techniques previously described by our laboratory [86]. Tissues were stained using the following primary antibodies: TNF-α (1:250; Abcam, ab6671), IL-10 (1:200; Santa Cruz Biotechnology SC-365858), nitrotyrosine (1:200; Santa Cruz Biotechnology, SC-32757) MOMA-2 (1:500, Abcam ab33451), and NF-κB p65 (1:500, Abcam, ab86299). Secondary antibodies used were anti-rabbit Alexa Fluor 555, anti-mouse Alexa Fluor 546, anti-mouse Alexa Fluor 555, anti-rabbit Alexa Fluor Plus 488, and anti-rat Alexa Fluor 488. Slides were imaged under fluorescent microscopy at 40x with the appropriate excitation/emission filter, digitally recorded, RGB overlay signals were split and analyzed for specific fluorescence using image densitometry with Image J software (NIH). A minimum of 3–4 locations on each section (4 sections per slide), 3 slides, and *n* = 3 per group were used for analysis; 40x images were used for image quantification.

### Real time RT-qPCR

Total RNA was isolated from the lungs (*n* = 6 per group) using an RNAEasy Mini kit (Qiagen, Valencia, CA) per kit instructions, and cDNA was synthesized using an iScript cDNA Synthesis kit (Biorad, Hercules, CA; Cat. #170–8891). Real-time PCR analysis of markers of inflammation TNF-α, IL-10, pIgR, FcRn, TLR2, TLR4, NF-κB p65, IL-1β, IL-6, as well as mucin genes Muc5b, Muc5ac, Muc4 was conducted using specific primers (Table 1) and SYBR green detection (Sso Advanced Universal SYBR Green Supermix, Biorad; Cat #172–5271), following manufacturer’s protocol. Samples were processed on a Biorad CFX96, and ΔΔC_T_ values calculated and normalized (to GAPDH), as previously described by our laboratory [89, 90].

**Table 1:** Mouse primer sequences used for RT-qPCR analysis

Muc5b FP:	5′ – CTGGCACCTGCTCTGTGCA – 3’
Muc5b RP:	5′ – CACTGCTTTGAGGCAGTTCT – 3’
Muc5ac FP:	5′ – ACGACACTTTTCAGTACCAATGAC – 3’
Muc5ac RP:	5′ – GCTTCCTTACAGATGCAGTCCT – 3’
pIgR FP:	5′ – AGTAACCGAGGCCTGTCCT – 3’
pIgR RP:	5′ – GTCACTCGGCAACTCAGGA – 3’
FcRn FP:	5′ – CCCTGGAGAAGATATTAAATGGGAC – 3’
FcRn RP:	5′ – TCAGGCTGCTTCATCCACAG – 3’
TNF-α FP:	5′ – CCACCACGCTCTTCTGTCTAC – 3’
TNF-α RP:	5′ – TGGGCTACAGGCTTGTCACT – 3’
NF-κB p65 FP:	5′ – CTTCCTCAGCCATGGTACCTCT – 3’
NF-κB p65 RP:	5′ – CAAGTCTTCATCAGCATCAAACTG – 3’
IL-1β FP:	5′ – CCTTCCAGGATGAGGACATGA – 3’
IL-1β RP:	5′ – TGAGTCACAGAGGATGG-GCTC – 3’
IL-6 FP:	5′ – CCGGAGAGGAGACTTCACAG – 3’
IL-6 RP:	5′ – GGAAATTGGGGTAGGAAGGA – 3’
IL-10 FP:	5′ – ATAACTGCACCCACTTCCCA – 3’
IL-10 RP:	5′ – GGGCATCACTTCTACCAGGT – 3’
TLR-2 FP:	5′ – GCCACCATTTCCACGGACT – 3’
TLR-2 RP:	5′ – GGCTTCCTCTTGGCCTGG – 3’
TLR-4 FP:	5′ – TTTATTCAGAGCCGTTGGTG – 3’
TLR-4 RP:	5′ – CAGAGGATTGTCCTCCCATT – 3’

### DNA isolation and qPCR

DNA from BALF and lung tissues (after the BALF was flushed) (*n* = 7 per group) were extracted using ZR Fecal DNA miniprep (Zymo Research). We generated E.coli bacterial standards and quantified 16S levels using Eubacteria primers. These standards were run on each plate and all the runs were performed on the same day. Cycling conditions were as follows: 95 °C for 4 min, followed by 45 cycles of 95 °C for 05 s, 55 °C for 1 min and 65 °C for 31 s, a final extension at 70 °C for 5 min. Data from the samples for each of the primers used were normalized to the Eubacteria E.coli standard**.** Bacterial primers used for Eubacteria, Firmicutes, Bacteroidetes and Proteobacteria and the method employed are described in ref. [91]. Primer sequences used for Actinobacteria are 5′ – CGCGGCCTATCAGCTTGTTG – 3′ and 5′ – CCGTACTCCCCAGGCGGGG - 3′. Data on controls are included in Supplementary Figure 3. DNA extraction was performed on all of the controls in Supplementary Figure 3 using the ZR Fecal DNA miniprep (Zymo Research).

### Elisa

IgA and IgG concentrations were measured using IgA (Fisher Scientific #EMIGA) and IgG (Fisher Scientific #88–50,400-22) in BALF (*n* = 8–10 per group) following the manufacturer’s recommended protocol. LPS ELISA was done using the mouse LPS kit (Cusabio, CSB-E13066m). ELISA was read on a Cytation5 plate reader at 450 nm absorbance. The samples were processed in triplicates for IgA and IgG concentrations and in duplicates for LPS measurements. The values were determined from a known value standard curve, using a sigmoidal four-parameter logistic (4-PL) curve-fit.

### Statistical analysis

Data were analyzed by two way ANOVA with Sidak-Holm multiple comparison all-pairwise test using GraphPad Prism 8. Two way ANOVA was used to determine the relationship between two independent factors - diet and exposure to DEP and the interaction between these two factors (reported as diet x. exposure interaction). For the probiotic studies, since all animals were on a high fat diet, a two way ANOVA was used to determine the statistical relationship between the two factors of exposure and probiotic-treatment, as well as the interaction between the two factors (reported as exposure x probiotic treatment). The corresponding *p* values and F values for each pairwise interaction are reported in the Results section for each endpoint. Data are expressed as mean ± SEM and a *p* < 0.05 was considered statistically significant.

## Supplementary Information

**Additional file 1: Supplemental Figure 1.** Probiotic supplementation alters the microbial profile. Quantification by qPCR of (A) Firmicutes, (B) Bacteroidetes, (C) Proteobacteria and (D) Actinobacteria within the lungs of C57Bl/6 wildtype mice on high-fat (HF) diet exposed to either saline (control) or diesel exhaust particles (DEP – 35 μg PM) twice a week for a total of 30 days alongside a dose of 0.3 g/day (~ 7.5 × 10^7^ cfu/day) of Ecologic® Barrier probiotics in the drinking water over the course of the exposures. Data are depicted as mean ± SEM with *p < 0.05 compared to HF Control, ‡*p* < 0.05 compared to HF DEP by two way ANOVA. **Supplemental Figure 2.** LPS Content in DEP material is negligible. Quantification of LPS (ng/ml) in the DEP material and saline used for the exposure study by ELISA. **Supplementary Figure 3.** Data on controls and DEP Material. (A) Quantification by qPCR of Eubacteria in PCR controls. (B) Nanodrop quantification of nucleic acid content in DEP and saline used for the exposure study. Data are depicted as mean ± SEM, *n* = 3–4 with *p < 0.001 compared to MB water - Plate Control, †*p* < 0.001 compared to DEP + Endotoxin free water, ‡*p* < 0.001 compared to DEP + Saline (used in the study), +*p* < 0.001 compared to Endotoxin free water control and ϕ*p* < 0.001 compared to saline by one way ANOVA with Sidak-Holm multiple comparison test.

## Data Availability

The datasets used and/or analyzed during the current study are available from the corresponding author on reasonable request.

## Abbreviations

BALF: Bronchoalveolar lavage fluid
COPD: Chronic obstructive pulmonary disorder
DEP: Diesel exhaust particulate matter
FcRn: Neonatal Fc receptor
HF: High-fat mouse chow
IgA: Immunoglobulin A
IgG: Immunoglobulin G
IL: Interleukin
IPF: Idiopathic pulmonary fibrosis
LF: Low-fat (standard) mouse chow
NF-κB: Nuclear factor kappa B
OA: Oropharyngeal aspiration
pIgR: Polymeric IgA receptor
ROS: Reactive oxygen species
RNS: Reactive nitrogen species
TLR-4: Toll-like receptor-4
TNF-α: Tumor necrosis factor alpha
T _reg_: T regulatory cells
