# Supplementary material for: Exposure to diesel exhaust particles results in altered lung microbial profiles, associated with increased reactive oxygen species/reactive nitrogen species and inflammation, in C57Bl/6 wildtype mice on a high-fat diet
Source: Part Fibre Toxicol. 2021 Jan 8;18:3. doi: 10.1186/s12989-020-00393-9 (PMC7796587; doi:10.1186/s12989-020-00393-9)
Supplement: Supplementary file 1 — Additional file 1: Supplemental Figure 1. Probiotic supplementation alters the microbial profile. Quantification by qPCR of (A) Firmicutes, (B) Bacteroidetes, (C) Proteobacteria and (D) Actinobacteria within the lungs of C57Bl/6 wildtype mice on high-fat (HF) diet exposed to either saline (control) or diesel exhaust particles (DEP – 35 μg PM) twice a week for a total of 30 days alongside a dose of 0.3 g/day (~ 7.5 × 10^7^ cfu/day) of Ecologic® Barrier probiotics in the drinking water over the course of the exposures. Data are depicted as mean ± SEM with *p < 0.05 compared to HF Control, ‡p < 0.05 compared to HF DEP by two way ANOVA. Supplemental Figure 2. LPS Content in DEP material is negligible. Quantification of LPS (ng/ml) in the DEP material and saline used for the exposure study by ELISA. Supplementary Figure 3. Data on controls and DEP Material. (A) Quantification by qPCR of Eubacteria in PCR controls. (B) Nanodrop quantification of nucleic acid content in DEP and saline used for the exposure study. Data are depicted as mean ± SEM, n = 3–4 with *p < 0.001 compared to MB water - Plate Control, †p < 0.001 compared to DEP + Endotoxin free water, ‡p < 0.001 compared to DEP + Saline (used in the study), +p < 0.001 compared to Endotoxin free water control and ϕp < 0.001 compared to saline by one way ANOVA with Sidak-Holm multiple comparison test. [file 12989_2020_393_MOESM1_ESM.pptx]

## Slide 1
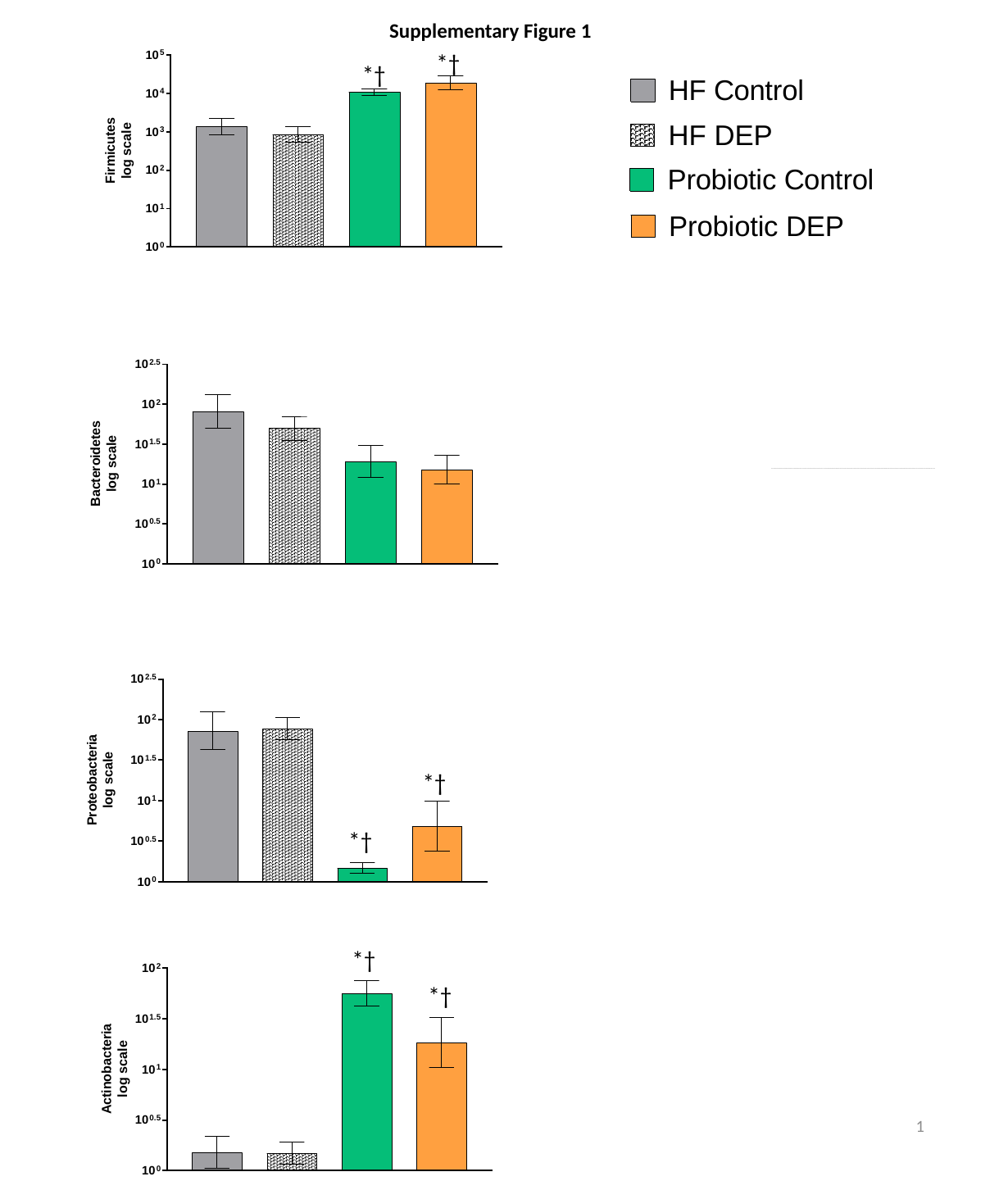

Supplementary Figure 1
*†
*†
*†
*†
*†
*†
1

## Slide 2
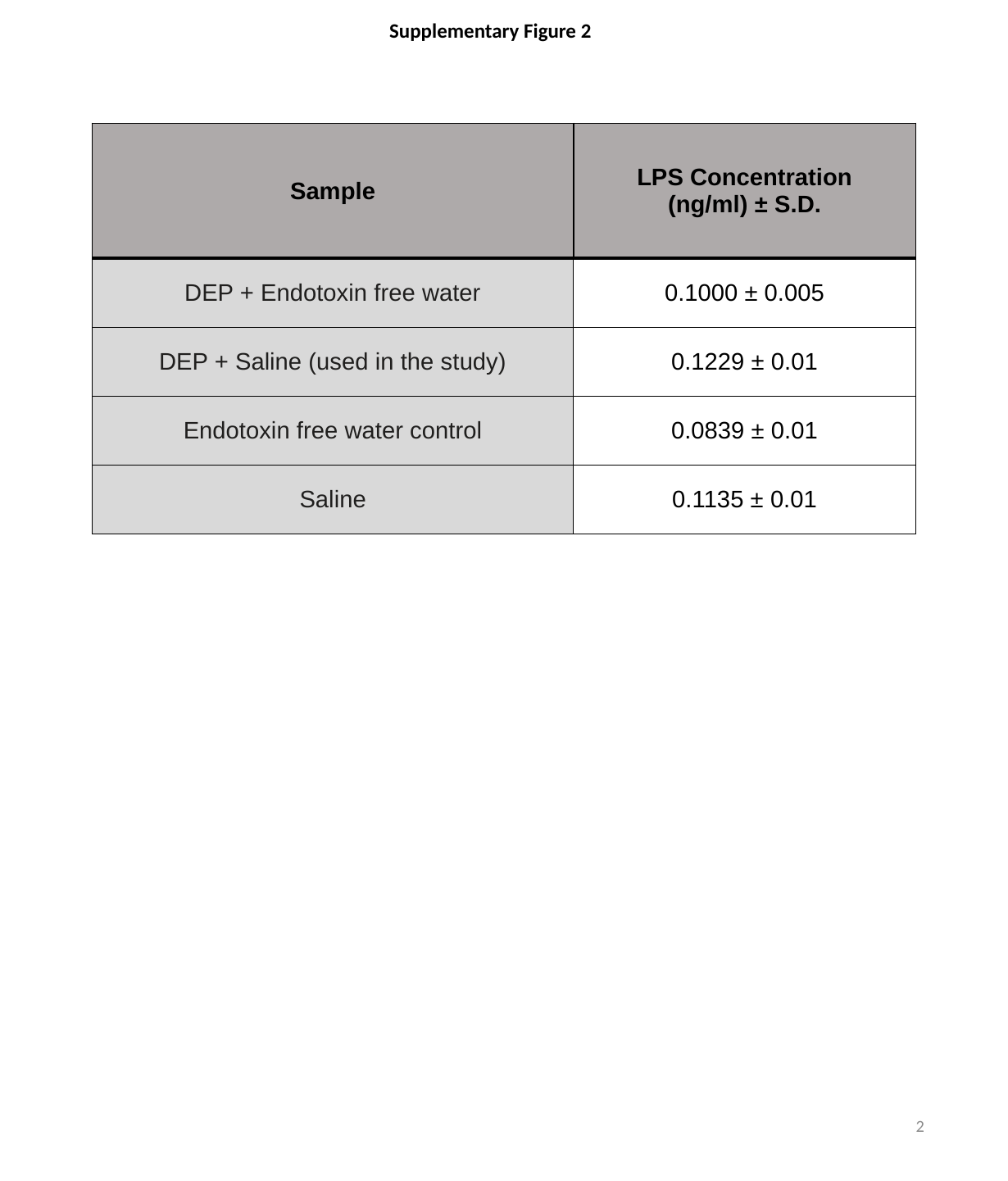

Supplementary Figure 2
| Sample | LPS Concentration (ng/ml) ± S.D. |
| --- | --- |
| DEP + Endotoxin free water | 0.1000 ± 0.005 |
| DEP + Saline (used in the study) | 0.1229 ± 0.01 |
| Endotoxin free water control | 0.0839 ± 0.01 |
| Saline | 0.1135 ± 0.01 |
2

## Slide 3
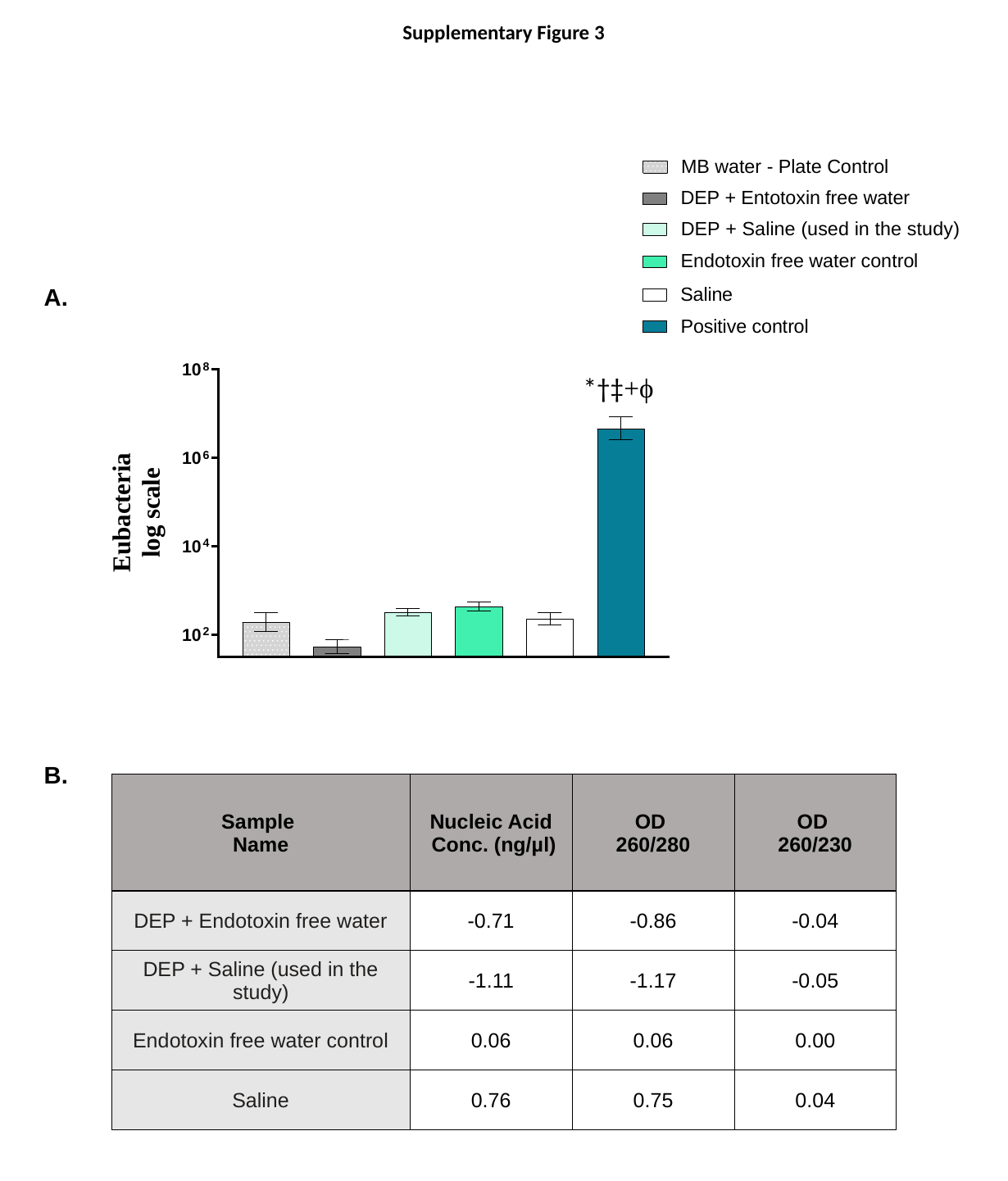

Supplementary Figure 3
A.
*†‡+ϕ
B.
| Sample Name | Nucleic Acid Conc. (ng/µl) | OD 260/280 | OD 260/230 |
| --- | --- | --- | --- |
| DEP + Endotoxin free water | -0.71 | -0.86 | -0.04 |
| DEP + Saline (used in the study) | -1.11 | -1.17 | -0.05 |
| Endotoxin free water control | 0.06 | 0.06 | 0.00 |
| Saline | 0.76 | 0.75 | 0.04 |
